# Experimental Nonalcoholic Steatohepatitis and Liver Fibrosis Are Ameliorated by Pharmacologic Activation of Nrf2 (NF-E2 p45-Related Factor 2)

**DOI:** 10.1016/j.jcmgh.2017.11.016

**Published:** 2017-12-13

**Authors:** Ritu S. Sharma, David J. Harrison, Dorothy Kisielewski, Diane M. Cassidy, Alison D. McNeilly, Jennifer R. Gallagher, Shaun V. Walsh, Tadashi Honda, Rory J. McCrimmon, Albena T. Dinkova-Kostova, Michael L.J. Ashford, John F. Dillon, John D. Hayes

**Affiliations:** 1 Division of Cancer Research, Ninewells Hospital and Medical School, University of Dundee, Dundee, Scotland, United Kingdom; 2 School of Medicine, University of St Andrews, St Andrews, Scotland, United Kingdom; 3 Division of Molecular and Clinical Medicine, Ninewells Hospital and Medical School, University of Dundee, Dundee, Scotland, United Kingdom; 4 Department of Pathology, Ninewells Hospital and Medical School, Tayside NHS Trust, Dundee, Scotland, United Kingdom; 5 Department of Chemistry and Institute of Chemical Biology & Drug Discovery, Stony Brook University, Stony Brook, New York

**Keywords:** NASH, Nrf2, TBE-31, ACACA, acetyl-CoA carboxylase alpha, ACLY, ATP citrate lyase, ACOT7, acetyl-CoA thioesterase 7, ACOX2, acetyl-CoA oxidase 2, ADRP, adipose differentiation-related protein, AP-1, activator protein 1, ApoB, apolipoprotein B, ATF4, activating transcription factor-4, ATF6, activating transcription factor-6, BCL-2, B-cell lymphoma, BIP, binding immunoglobulin protein, CAT, catalase, CD36, cluster of differentiation 36, CDDO, 2-cyano-3,12-dioxooleana-1,9(11)-dien-28-oic acid, C/EBP, CCAAT/enhancer-binding protein, CES1G, carboxylesterase 1g, CHOP, C/EBP homologous protein, ChREBP, carbohydrate-responsive element-binding protein, COL1A1, collagen, type I, alpha-1, COX2, cyclooxygenase-2, CPT1A, carnitine palmitoyltransferase 1a, DGAT2, diacylglycerol acyltransferase-2, DMSO, dimethyl sulfoxide, eIf2α, eukaryotic translation initiation factor 2A, ER, endoplasmic reticulum, FASN, fatty acid synthase, FXR, farnesoid X receptor, GCLC, glutamate-cysteine ligase catalytic, GCLM, glutamate-cysteine ligase modifier, GPX2, glutathione peroxidase-2, GSH, reduced glutathione, GSSG, oxidized glutathione, GSTA4, glutathione *S-*transferase Alpha-4, GSTM1, glutathione *S-*transferase Mu-1, GTT, glucose tolerance test, H&E, hematoxylin and eosin, HF, high-fat, HFFr, high-fat diet with fructose in drinking water, HF30Fr, high-fat diet with 30% fructose in drinking water, HF55Fr, high-fat diet with 55% fructose in drinking water, HMOX1, heme oxygenase-1, IRE1α, inositol requiring kinase-1α, IκB, inhibitor of NF-κB, IKK, IκB kinase, ITT, insulin tolerance test, JNK1, c-Jun N-terminal kinase 1, Keap1, Kelch-like ECH-associated protein-1, LXRα, liver X receptor α, MCD, methionine- and choline-deficient, MCP-1, monocyte chemotactic protein-1, MGPAT, mitochondrial glycerol-3-phosphate acetyltransferase, MPO, myeloperoxidase, MTTP, microsomal triglyceride transfer protein, NAFLD, non-alcoholic fatty liver disease, NAS, NAFLD activity score, NASH, nonalcoholic steatohepatitis, NF-κB, nuclear factor-κB, NOS2, nitric oxide synthase-2, NQO1, NAD(P)H:quinone oxidoreductase 1, Nrf2, NF-E2 p45-related factor 2, p58^IPK^, p58 inhibitor of the PKR kinase, PARP, poly ADP ribose polymerase, PCR, polymerase chain reaction, PDI, protein disulfide isomerase, PERK, PRK-like endoplasmic reticulum kinase, PPARα, peroxisome proliferator-activated receptor α, PPARγ, peroxisome proliferator-activated receptor γ, PRDX6, peroxiredoxin 6, PTGR1, prostaglandin reductase-1, PTT, pyruvate tolerance test, qRT-PCR, quantitative reverse transcriptase PCR, RC, regular chow, SCAD, short-chain acyl-CoA dehydrogenase, SCD1, stearoyl-CoA desaturase-1, SFN, sulforaphane, SHP, small heterodimer partner, SLC7A11, solute carrier family 7 member 11, α-SMA, alpha smooth muscle actin, SREBP-1c, sterol regulatory element-binding protein-1c, TGFβ, transforming growth factor beta-1, TNF-α, tumor necrosis factor-α, TXN1, thioredoxin-1, TXNRD1, thioredoxin reductase-1, UPR, unfolded protein response, XBP1, X-box binding protein-1

## Abstract

**Background & Aims:**

Nonalcoholic steatohepatitis (NASH) is associated with oxidative stress. We surmised that pharmacologic activation of NF-E2 p45-related factor 2 (Nrf2) using the acetylenic tricyclic bis(cyano enone) TBE-31 would suppress NASH because Nrf2 is a transcriptional master regulator of intracellular redox homeostasis.

**Methods:**

*Nrf2*^*+/+*^ and *Nrf2*^*-/-*^ C57BL/6 mice were fed a high-fat plus fructose (HFFr) or regular chow diet for 16 weeks or 30 weeks, and then treated for the final 6 weeks, while still being fed the same HFFr or regular chow diets, with either TBE-31 or dimethyl sulfoxide vehicle control. Measures of whole-body glucose homeostasis, histologic assessment of liver, and biochemical and molecular measurements of steatosis, endoplasmic reticulum (ER) stress, inflammation, apoptosis, fibrosis, and oxidative stress were performed in livers from these animals.

**Results:**

TBE-31 treatment reversed insulin resistance in HFFr-fed wild-type mice, but not in HFFr-fed Nrf2-null mice. TBE-31 treatment of HFFr-fed wild-type mice substantially decreased liver steatosis and expression of lipid synthesis genes, while increasing hepatic expression of fatty acid oxidation and lipoprotein assembly genes. Also, TBE-31 treatment decreased ER stress, expression of inflammation genes, and markers of apoptosis, fibrosis, and oxidative stress in the livers of HFFr-fed wild-type mice. By comparison, TBE-31 did not decrease steatosis, ER stress, lipogenesis, inflammation, fibrosis, or oxidative stress in livers of HFFr-fed Nrf2-null mice.

**Conclusions:**

Pharmacologic activation of Nrf2 in mice that had already been rendered obese and insulin resistant reversed insulin resistance, suppressed hepatic steatosis, and mitigated against NASH and liver fibrosis, effects that we principally attribute to inhibition of ER, inflammatory, and oxidative stress.

## Graphical abstract

**Figure:**
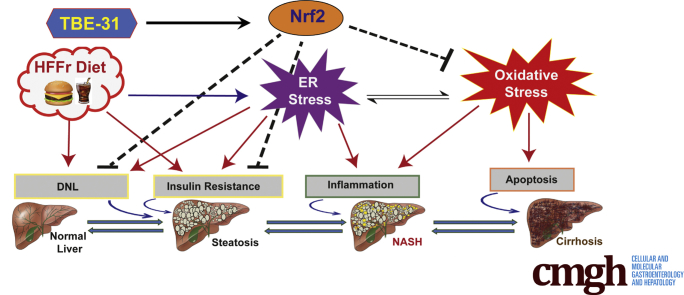

**Summary**

In mice with diet-stimulated nonalcoholic steatohepatitis, pharmacologic activation of transcription factor Nrf2 improves glucose homeostasis and inhibits hepatic steatosis, inflammation, and fibrosis. Nrf2-mediated amelioration of nonalcoholic steatohepatitis and liver fibrosis involves downregulation of lipogenic genes, induction of antioxidant genes, and suppression of both oxidative and endoplasmic reticulum stress.

Nonalcoholic fatty liver disease (NAFLD) is associated with type-2 diabetes mellitus, insulin resistance, and obesity, as well as chronic overconsumption of an energy-dense diet containing high-fat (HF) food and sweetened beverages that contain fructose.^1^ It comprises a spectrum of phenotypes ranging from simple steatosis to nonalcoholic steatohepatitis (NASH).^2^ NAFLD is a major health concern because between 20% and 40% of adults who consume a western-style diet have NAFLD, of which approximately 15% suffer NASH. In some individuals, NASH progresses to cirrhosis and hepatocellular carcinoma.^3^

The development of NASH entails the presence of insulin resistance, and increases in *de novo* lipogenesis, inflammation, and oxidative stress.^4^, ^5^ The relationship between simple steatosis and NASH can be viewed as a dynamic one, with steatosis representing successful adaptation to metabolic stress, and NASH reflecting failure on the part of mitochondria to adapt adequately to an increased metabolic burden, which in turn leads to increased mitochondrial production of reactive oxygen species.^6^, ^7^ Although NASH is principally a disease of hepatocytes, the gut and adipose tissue also contribute to hepatic insulin resistance and inflammation.^4^

The appearance of insulin resistance, *de novo* lipogenesis, inflammation, and oxidative stress during the development of NASH seems intertwined and each is linked to endoplasmic reticulum (ER) dysfunction.^4^, ^8^ Aberrant protein folding within the ER, which represents ER stress, stimulates the unfolded protein response (UPR) through activation of 3 pathways, controlled by inositol requiring kinase-1α (IRE1α), activating transcription factor-6 (ATF6), and PRK-like ER kinase (PERK), and together these initiate an adaptive program that serves to restore proteostasis.^9^ However, if ER stress persists in the liver for a prolonged period, it produces pathophysiological changes associated with NASH. For example, chronic ER stress can result in insulin resistance through stimulation of a pathway downstream of IRE1α that leads to increased serine phosphorylation of insulin receptor substrate-1.^10^ Also, persistent activation of the UPR stimulates hepatic *de novo* lipogenesis via all 3 arms of the UPR, causing increased activity of sterol regulatory element-binding protein-1c (SREBP-1c, encoded by *SREBF1*), carbohydrate-responsive element-binding protein (ChREBP, encoded by *MLXIPL*), the spliced variant of X-box binding protein-1 (XBP1s), peroxisome proliferator-activated receptor gamma (PPARγ), CCAAT/enhancer-binding protein (C/EBP) α and C/EBPβ,^11^, ^12^, ^13^ and in turn increased lipogenesis exacerbates insulin resistance. Moreover, chronic ER stress triggers inflammation via IRE1α, resulting in stimulation of IκB kinase (IKK) and c-jun *N-*terminal protein kinase 1 (JNK1) that activate nuclear factor-κB (NF-κB) and activator protein 1 (AP-1), respectively,^14^ events that are also linked to insulin resistance.^4^, ^8^ It may also stimulate inflammation via PERK by attenuating translation of the inhibitor of NF-κB (IκB), which results in a relative excess of NF-κB.^15^, ^16^ Lastly, chronic ER stress can initiate oxidative stress by augmenting both oxidoreductin-1 activity and release of Ca^2+^ from the ER, which in turn heighten mitochondrial production of reactive oxygen species,^17^ and so sensitizes the innate immune system to proinflammatory stimuli.^18^ Thus, chronic ER stress promotes insulin resistance, lipogenesis, inflammation, and oxidative stress.

Because oxidative stress contributes to NASH, researchers have examined whether loss of NF-E2 p45-related factor 2 (Nrf2, encoded by *NFE2L2*) increases susceptibility to the disease because Nrf2 is a master regulator of cellular redox homeostasis that orchestrates adaptation to intracellular redox perturbation.^19^ Consistent with the view that oxidative stress is pivotal in development of NASH, knockout of Nrf2 in mice profoundly predisposes to NASH stimulated by either a methionine- and choline-deficient (MCD) diet^20^, ^21^ or a HF diet.^22^, ^23^, ^24^ Although loss of Nrf2 increases sensitivity to NASH, it is less certain whether upregulation of Nrf2 by genetic or pharmacologic approaches decreases sensitivity to the disease. Specifically, genetic activation of Nrf2 in mice by knockdown of its repressor Kelch-like ECH-associated protein-1 (Keap1) has been reported to inhibit liver steatosis and NASH caused by an MCD diet,^25^, ^26^ but genetic activation of Nrf2 in mice by knockdown of Keap1 has also been reported to increase NASH caused by a HF diet^27^ and to increase insulin resistance and liver steatosis when crossed onto a *Lep*^*ob/ob*^ background.^28^ Set against these seemingly discrepant results are several studies showing that pharmacologic activation of Nrf2 protects against diabetes and NAFLD: these include the findings that treatment with the triterpenoid 2-cyano-3,12-dioxooleana-1,9(11)-dien-28-oic acid (CDDO)-methyl ester (bardoxolone methyl) ameliorates diabetes and hepatic steatosis in HF-fed mice^29^, ^30^ and that treatment with CDDO-imidazole attenuates diabetes in *Lepr*^*db/db*^ mice.^31^ The possibility that treatment with Nrf2 activators after the onset of diabetes might reverse insulin resistance along with advanced stages of NAFLD has received little attention hitherto.

In the present study, we tested whether pharmacologic activation of Nrf2 suppresses NASH and have examined if this can happen after disease is manifest because this scenario reflects the clinical situation. We therefore stimulated NASH by feeding mice chronically with a HF diet along with fructose-containing drinking water (called HFFr diet). After the high-calorie regimen had been in place for 24 weeks (Study 1), and mice exhibited impaired glucose tolerance and hyperinsulinemia, we used the potent Nrf2 activator TBE-31^32^, ^33^ (Figure 1) to test if it could improve insulin sensitivity and glucose homeostasis, and mitigate NASH in the liver. Lastly, we used *Nrf2*^*-/-*^ mice (Study 2) to demonstrate that the beneficial effects of TBE-31 in this setting require the presence of Nrf2.

**Figure 1:**
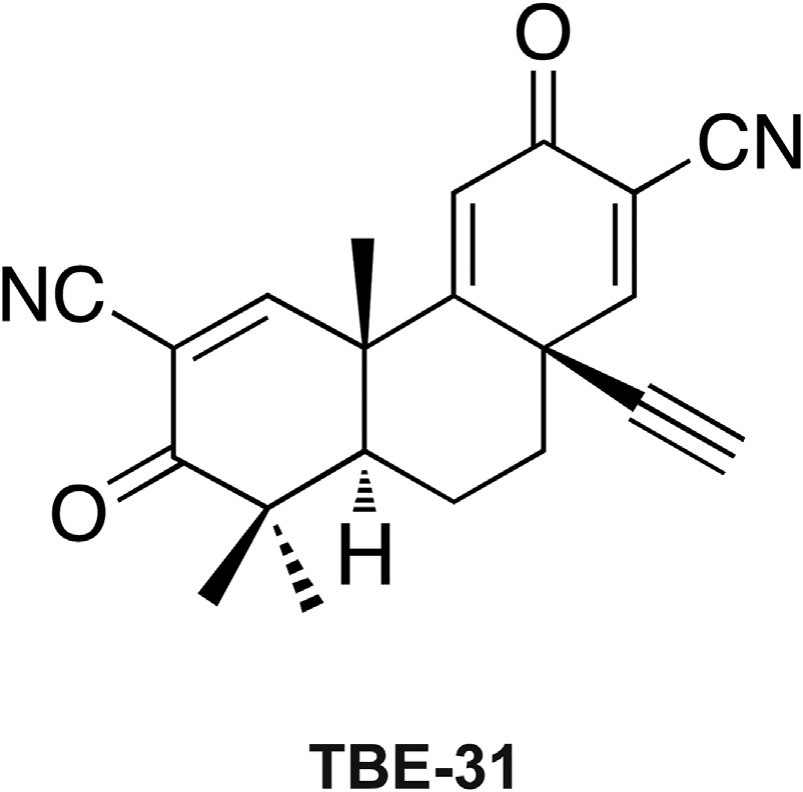
**Structure of TBE-31**.

## Materials and Methods

### Chemicals

Unless otherwise stated, these were from Sigma-Aldrich (St. Louis, MO). The acetylenic tricyclic bis(cyano enone) compound TBE-31 was synthesized as described previously.^32^, ^34^

### Animals

Male C57BL/6 mice were purchased from Charles River (Elphinstone, Tranent, Scotland, UK) and allowed to acclimatize for 2 weeks before being fed specific diets. *Nrf2*^*-/-*^ mice,^35^ backcrossed over at least 7 generations onto a C57BL/6 background, were also studied. All animal care protocols and experimental procedures were performed in accordance with the Animal Scientific Procedures Act (1986) and with approval of the local University of Dundee Animal Ethics Committee. From 8 to 10 weeks of age, the mice were provided *ad libitum* either regular chow (RC; containing 7.5% fat by energy) or a HF diet (containing 60% fat by energy), purchased from SDS Ltd (Witham, Essex, UK). Animals were killed by cervical dislocation or by terminal anesthesia using isofluorane. On sacrifice, livers were removed immediately and portions rapidly snap-frozen in liquid N_2_ (and subsequently stored at -80°C) or fixed in formalin as described elsewhere.^20^

All our experiments were performed in mice on a C57BL/6 background because this strain is widely used to study obesity and insulin resistance.^36^ It is, however, well recognized that a proportion of these mice do not become obese when fed a HF diet, for a variety of reasons, including failure to become diabetic and increased metabolic adaptation resulting in an inability to saturate the trichloroacetic acid cycle and mitochondrial oxidative metabolism.^37^, ^38^ Consistent with this previously documented characteristic of the C57BL/6 mouse, we found a small number of animals did not become obese when fed the HF55Fr/HF30Fr diet, and these were segregated from the others as nonresponders, and the remaining obese mice were randomized into 2 different treatment groups (see Results section).

To ensure rapid development of NASH in wild-type C57BL/6 mice, the HF diet was initially provided along with drinking water containing 55% (wt/vol) fructose (ie, the HF55Fr diet), which was used to prime *Nrf2*^*+/+*^ mice for NASH before attenuating the rate of disease development by placing them on the HF diet along with drinking water containing 30% (wt/vol) fructose (ie, the standard HF30Fr diet). The sequential provision of HF55Fr and HF30Fr diets, given only to *Nrf2*^*+/+*^ C57BL/6 mice, is described in the text as the HF55Fr/HF30Fr diet. For dietary challenge of *Nrf2*^*-/-*^ mice, only the standard HF30Fr diet was provided because they are more sensitive to NASH (when placed on a HF diet)^24^: because of this innate sensitivity, we thought it unnecessary to prime *Nrf2*^*-/-*^ mice by placing them on a HF55Fr diet.

**Figure 2:**
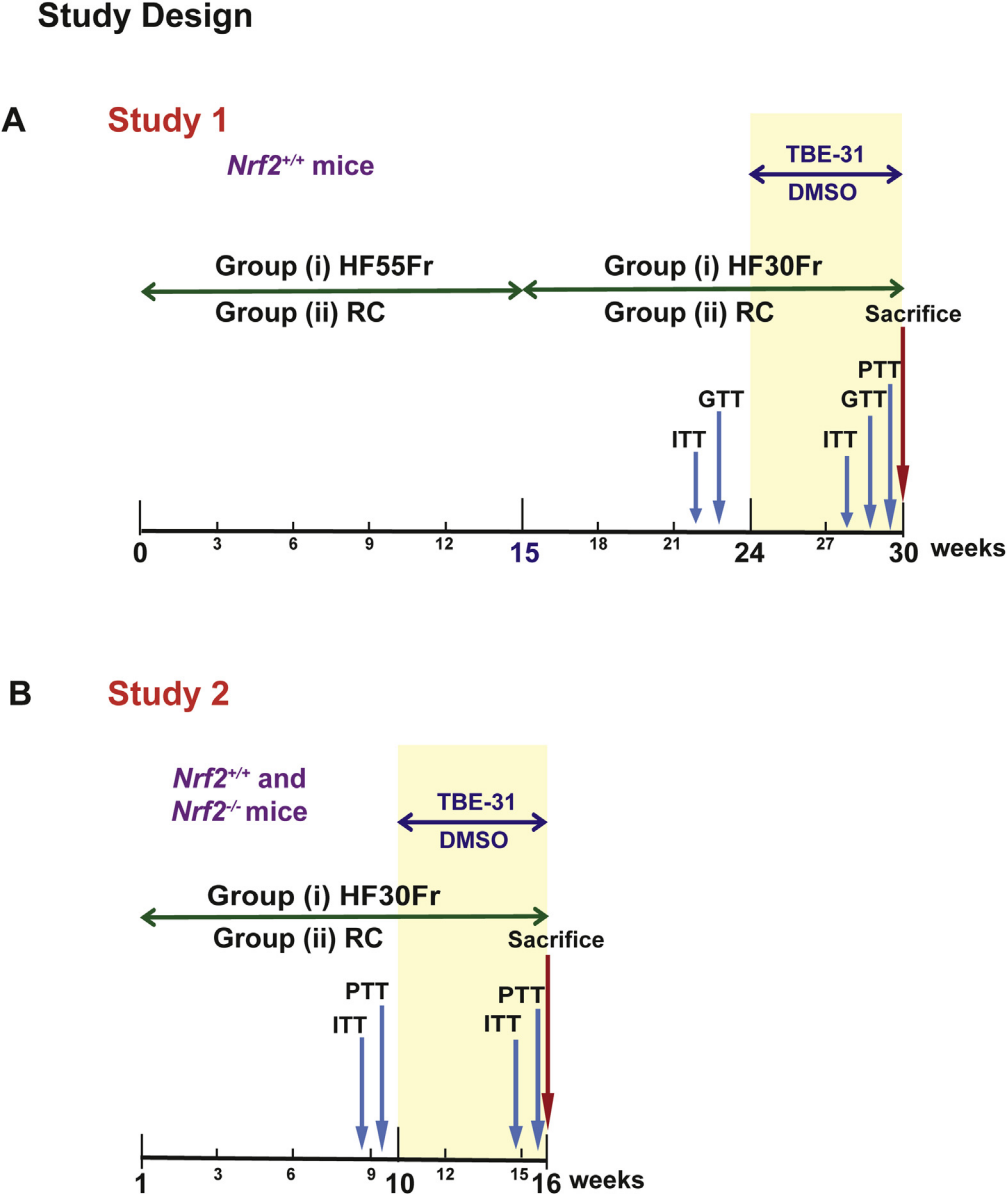
**Experimental design.** (*A*, Study 1). In group (i), *Nrf2*^*+/+*^ C57BL/6 mice were first primed over a period of 15 weeks for NASH by feeding a HF55Fr diet before being transferred to the standard HF30Fr diet at the beginning of Week 16. In group (ii), an equal number of age-matched *Nrf2*^*+/+*^ mice were fed an RC diet along with unadulterated drinking water throughout. After being placed for 24 weeks on either of these 2 dietary regimens, mice in group (i) and group (ii) were treated with either TBE-31 (5 nmol/g body weight) or DMSO vehicle control, by oral gavage 3 times/week for a total of 6 weeks, while still being provided with the same HF30Fr diet or RC diet. Glucose homeostasis was monitored in all mice by ITT, GTT, and PTT at the times indicated. (*B*, Study 2). In group (i), *Nrf2*^*+/+*^ and *Nrf2*^*-/-*^ C57BL/6 mice, of 8–10 weeks of age, were fed the standard HF30Fr diet for 10 weeks before being treated with either TBE-31 or DMSO for a total of 6 weeks while being maintained on the same diet. In group (ii), *Nrf2*^*+/+*^ and *Nrf2*^*-/-*^ mice were fed the RC diet for 10 weeks, with no fructose in the drinking water, before being treated with either TBE-31 or DMSO for a further 6 weeks while being maintained on the same diet. Glucose homeostasis was monitored in all mice by ITT and PTT at the times indicated.

To test whether pharmacologic activation of Nrf2 inhibits the progression of NASH, 2 experimental protocols were adopted, with different objectives. In both cases, TBE-31 was administered (at 5 nmol/g body weight) after insulin resistance had been established, and while the animals were still receiving the HF30Fr diet: the objective of Study 1 was to test whether activation of Nrf2 by TBE-31 could reverse insulin resistance and suppress NAFLD once disease was established; the objective of Study 2 was to test whether the ability of TBE-31 to reverse insulin resistance and suppress NAFLD requires the presence of Nrf2. In Study 1, *Nrf2*^*+/+*^ mice were initially primed for 15 weeks with the HF55Fr diet, followed by a further 9 weeks on the standard HF30Fr diet before treatment with TBE-31, or dimethyl sulfoxide (DMSO) vehicle control, 3 times a week for a total of 6 weeks by oral gavage while still being fed the standard HF30Fr diet (Figure 2*A*). In Study 2, *Nrf2*^*-/-*^ and *Nrf2*^*+/+*^ mice were provided with the standard HF30Fr diet for 10 weeks, before being treated with TBE-31, or DMSO, for a further 6 weeks while continuing on the same diet (Figure 2*B*). In both Study 1 and Study 2, mice were weighed weekly throughout the experiments and were killed 20–24 hours after receiving the final dose of TBE-31 or DMSO by gavage. On sacrifice, blood was collected from the mice and their livers removed immediately for histologic, biochemical, and molecular analyses.

### Physiological and Clinical Chemistry Measurements

The glucose tolerance test (GTT), insulin tolerance test (ITT), and pyruvate tolerance test (PTT) were carried out by intraperitoneal administration of standard doses of glucose, insulin, or pyruvate.^20^, ^24^ For Study 1, an ITT and GTT were first performed on the mice 1.5−2.5 weeks immediately preceding treatment with TBE-31, and were repeated when the mice had been treated for 4−5 weeks with TBE-31 (during Weeks 28 and 29 of Study 1). Subsequently, a single PTT was performed on these mice during Week 30. For Study 2, an ITT and PTT were first performed on mice 5−10 days immediately preceding treatment with TBE-31, and were repeated when they had been treated for 4−5 weeks with TBE-31 (during Weeks 14 and 15 of Study 2). Blood samples were collected via the tail vein or via cardiac puncture. Blood glucose, triglycerides, cholesterol, and plasma leptin and insulin were measured as described previously.^24^ Fasting blood glucose and insulin levels were measured after diet and drinking water had been withdrawn for 5 hours. Plasma alanine aminotransferase activity was measured using commercial kits.^24^

### Histology

Mouse liver samples were fixed in 10% neutral buffered formaldehyde and processed for hematoxylin and eosin (H&E) and van Gieson staining by standard methods. The severity of liver disease in mice was evaluated histologically on H&E-stained sections using the NAFLD activity score (NAS)^39^ in a blinded fashion, in which the pathologists were unaware of the diet, treatment group, or genotype of the mice.

### Antibodies

Antibodies against proliferating cell nuclear antigen, binding immunoglobulin protein (BIP), protein disulfide isomerase (PDI), activating transcription factor-4 (ATF4), JNK, phospho-JNK, phospho-eukaryotic translation initiation factor 2A (eIf2α), p58 inhibitor of the PKR kinase (p58^IPK^), poly ADP ribose polymerase (PARP), caspase-3, caspase-9, NF-κB subunits p50, p52 and p65, IκB and phospho-IKK were purchased from Cell Signaling Technology (Danvers, MA). Antibody against actin was from Sigma-Aldrich, antibody against SREBP-1c was from Millipore (Burlington, MA), and that against ATF6 was from Santa Cruz Biotechnology (Dallas, TX). Antibody against XBP1 was from Abcam and that against phospho-Ire1α from ThermoFisher Scientific.

### Biochemical Analyses

Frozen mouse livers (about 100 mg each) were pulverized under liquid nitrogen using a pestle and mortar. The ground material was resuspended in ice-cold RIPA lysis buffer to which had been added protease and phosphatase inhibitors (Roche) before homogenization. Whole-cell lysates used for Western blot analyses represented the supernatant fraction (15,000 × *g*, 15 min at 4°C) obtained from the ground hepatic extracts. For the Nfkb and Srebp-1c Western blots, nuclear and cytoplasmic fraction extracts were prepared from frozen liver using the Pierce NE-PER kit (ThermoScientific Life Science Research Products). Protein concentrations were determined using bicinchoninic acid, and assays for NAD(P)H:quinone oxidoreductase-1 (Nqo1) enzyme activity toward menadione, total glutathione, reduced glutathione (GSH), oxidized glutathione (GSSG), and malondialdehyde were performed as described previously.^20^, ^40^

### Gene Expression Profiling

Total RNA was extracted from frozen mouse liver using the RNeasy kit (Qiagen, Hilden, Germany), and cDNA prepared using the Omniscript kit (Qiagen) according to the manufacturer’s instructions. The relative abundance of hepatic mRNA species was measured against actin as an internal control by TaqMan real-time polymerase chain reaction (PCR; Applied Biosystems Prism model 7700) using commercial primer and probe sets (Table 1), all purchased from Life Technologies.

**Table 1:** qRT-PCR Primers and Probe Sets

Gene	Protein encoded	Assay ID
*Acaca*	Acetyl-CoA carboxylase 1	Mm01304257_m1
*Acly*	ATP Citrate Lyase	Mm01302282_m1
*Acot7*	Acyl-CoA Thioesterase 7	Mm00460107_m1
*Acox2*	Acyl-CoA Oxidase 2	Mm00446408_m1
*ApoB*	Apolipoprotein B	Mm01545150_m1
*Adrp*	Adipose differentiation related protein	Mm00475794_m1
*Atf4*	Activating Transcription Factor 4	Mm00515325_g1
*ɑSma*	actin, alpha 2, smooth muscle, aorta	Mm00725412_s1
*Bcl2*	B cell leukemia/lymphoma 2	Mm00477631_m1
*Cat*	Catalse	Mm00437992_m1
*Cd36*	Cluster of differentiation 36	Mm01135198_m1
*Ces1g*	Carboxylesterase 1 g	Mm00491334_m1
*Chop*	CCAAT/enhancer-binding protein homologous protein	Mm01135937_g1
*Col1a1*	Collagen type I alpha 1 chain	Mm00801666_g1
*Cox2*	Cyclooxygenase-2	Mm03294838_g1
*Cpt1a*	Carnitine palmitoyltransferase 1A	Mm01231183_m1
*Dgat1*	Diacylglycerol O-acyltransferase 1	Mm00515643_m1
*Dgat2*	Diacylglycerol O-acyltransferase 2	Mm00499536_m1
*Elastase*	Elastase	Mm00712898_m1
*Fasn*	Fatty acid synthase	Mm00662319_m1
*Gclc*	Glutamate-cysteine ligase catalytic subunit	Mm00802655_m1
*Gclm*	Glutamate-cysteine ligase modifier subunit	Mm01324400_m1
*Gpx2*	Glutathione peroxidase 2	Mm00850074_g1
*Gsta4*	Glutathione *S-*transferase alpha 4	Mm00494803_m1
Gstm1	Glutathione *S-*transferase mu 1	Mm00833915_g1
*Hmox1*	Heme oxygenase 1	Mm00516005_m1
*Ifng*	Interferon gamma	Mm01168134_m1
*Il1b*	Interleukin 1 beta	Mm00434228_m1
*Lipin1*	Lipin 1	Mm00550511_m1
*Lxrɑ*	Nuclear receptor subfamily 1 group H member 3	Mm00443451_m1
*Mcp1*	Monocyte chemotactic protein 1	Mm00441242_m1
*Mgpat*	1-acylglycerol-3-phosphate O-acyltransferase 9	Mm04211965_m1
*Mixipl*	MLX interacting protein-like (also known as Chrebp)	Mm02342723_m1
*Mmp9*	Matrix metallopeptidase 9	Mm00442991_m1
*Mpo*	Myeloperoxidase	Mm01298424_m1
*Mttp*	Microsomal triglyceride transfer protein	Mm01321412_g1
*Nos2*	Nitric oxide synthase 2	Mm00440495_g1
*Nqo1*	NAD(P)H:quinone oxidoreductase 1	Mm01253561_m1
*Perk*	Eukaryotic translation initiation factor 2 alpha kinase 3	Mm00438700_m1
*Pparɑ*	Peroxisome proliferator-activated receptor alpha	Mm00440939_m1
*Prdx6*	Peroxiredoxin 6	Mm00725435_s1
*Ptgr1*	Prostaglandin reductase 1	Mm00482476_m1
*Scad*	Short-chain acyl-CoA dehydrogenase	Mm00431617_m1
*Scd1*	Stearoyl-Coenzyme A desaturase 1	Mm00772290_m1
*Slc7a11*	Solute carrier family 7 member 11	Mm01292536_m1
*Srebf1*	Sterol regulatory element binding transcription factor 1	Mm00550338_m1
*Tgfb*	Transforming growth factor beta 1	Mm01178820_m1
*Tnfɑ*	Tumor necrosis factor alpha	Mm00443258_m1
*Txn1*	Thioredoxin 1	Mm00726847_s1
*Txnrd1*	Thioredoxin reductase 1	Mm00443675_m1

qRT-PCR, quantitative reverse transcriptase polymerase chain reaction.

### Western Blotting

The electrophoretic resolution of proteins was performed using precast NuPAGE Bis-Tris gels from Invitrogen (ThermoFisher Scientific). Following transfer to polyvinylidene difluoride membranes, the immunoblots were developed with an electrochemiluminescence-based system (Millipore) according to the manufacturer’s protocols. Film was used to allow visualization of cross-reacting proteins; several exposures were tested before choosing the exposure that permitted detection of bands in all lanes. Quantification of the immunoblot data was conducted by measuring the band intensities using ImageJ software, which is freely available. In Study 1, immunoblotting was performed on liver samples prepared from 6 different mice for each of the diet and treatment groups. In Study 2, immunoblotting was performed on liver samples prepared from 4 different mice for each of the genotypes, diet, and treatment groups.

### Statistics

Comparisons between the biochemical and molecular biology results from the experimental groups were made using Student *t* test or Mann-Whitney test. Data for GTT, ITT, and PTT were analyzed by 1- or 2-way analysis of variance with Bonferroni postcorrection. When appropriate, repeated analysis of variance was performed with time as subject factor. Results presented are means ± SEM. On all occasions, data from RC-fed *Nrf2*^*+/+*^ mice that had been treated with DMSO vehicle control were used as the reference point, with significant increases being indicated by asterisk symbols and decreases associated with TBE-31 treatment indicated by dollar symbols, depending on the degree of significance (corrected *P* values ≤ .05 considered statistically significant); in addition, significant differences between data from HFFr-fed mice and other data sets are indicated by a horizontal bar that is placed above the relevant groups. Comparisons between the histology NAFLD activity scores were made using the Kruskal-Wallis H test.

## Results

### TBE-31 Improves Insulin Sensitivity and Diminishes Histologic Evidence of Nonalcoholic Steatohepatitis and Cirrhosis

**Figure 3:**
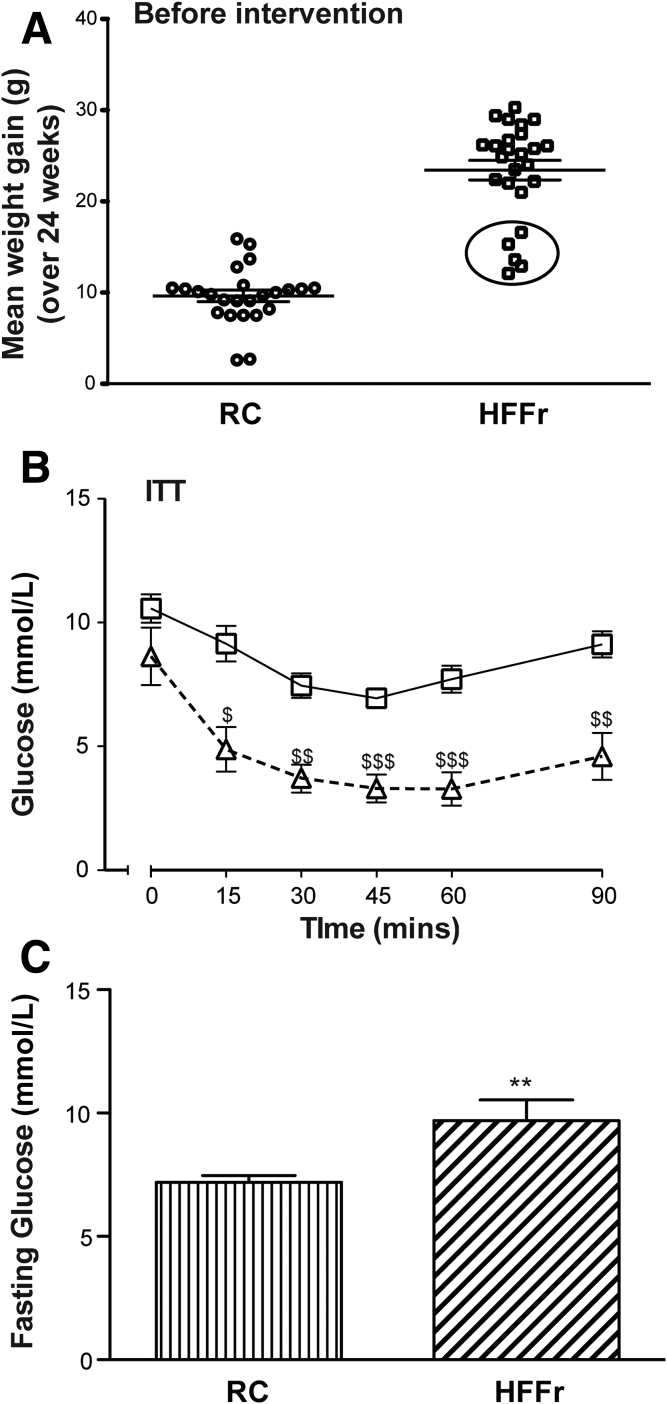
***Nrf2***^***+/+***^**mice become obese when fed the HFFr diet, and this is associated with hyperglycemia.***Nrf2*^*+/+*^ mice were fed either the RC or the HF55Fr/HF30Fr (HFFr) diet for 24 weeks. Before treatment with TBE-31 or DMSO control, the physiological effect of these dietary regimens was assessed. (*A*) Weight gain of individual mice over the 24-week period on the RC or HFFr diets is presented. The *encircled mice*, shown at the bottom of the HFFr plot, were excluded from the study on the basis that they failed to become obese.^36^, ^37^, ^38^ (*B*) A comparison of insulin sensitivity (ITT at 22 weeks) of the 5 nonobese *encircled* unresponsive HFFr-fed mice (*triangles*) with that of the 20 obese responsive HFFr-fed mice (*squares*). Results are means ± SEM (n = 5 or 20 mice, for nonobese and obese groups, respectively), and significant decreases in blood glucose in the nonobese unresponsive mice compared with the obese responsive mice are indicated by: ^$^*P* < .05; ^$$^*P* < .01; ^$$$^*P* < .001. (*C*) The fasting blood glucose levels of *Nrf2*^*+/+*^ mice fed RC or HFFr diets. Results are means ± SEM (n = 8–12 mice per group). Significant increases in fasting blood glucose, relative to that in RC-fed *Nrf2*^*+/+*^ mice, are indicated by: **P < .01.

In Study 1, NASH was produced in wild-type C57BL/6 mice using the HF55Fr/HF30Fr diet. After 24 weeks on this regimen (Figure 2*A*), *Nrf2*^*+/+*^ mice were approximately 2.3-fold heavier than age-matched RC-fed animals. At this time point, 20 of the 25 HF55Fr/HF30Fr-fed mice were obese, having gained 21.0−30.3 g weight over the 24-week period, whereas the remaining 5 gained 12.1−16.6 g weight and were judged to be nonobese (Figure 3*A*). The 5 nonobese HF55Fr/HF30Fr-fed mice, which had not gained more weight than the RC-fed mice over the same period, were excluded from further study because they were considered to be nonresponders^37^, ^38^; interestingly, ITT analyses revealed these nonresponders cleared glucose from the blood more effectively than did the remaining 20 obese mice, suggesting they were sensitive to insulin (Figure 3*B*). Among the remaining animals, fasting blood glucose concentrations were greater in HF55Fr/HF30Fr-fed mice than in RC-fed mice (Figure 3*C*).

**Figure 4:**
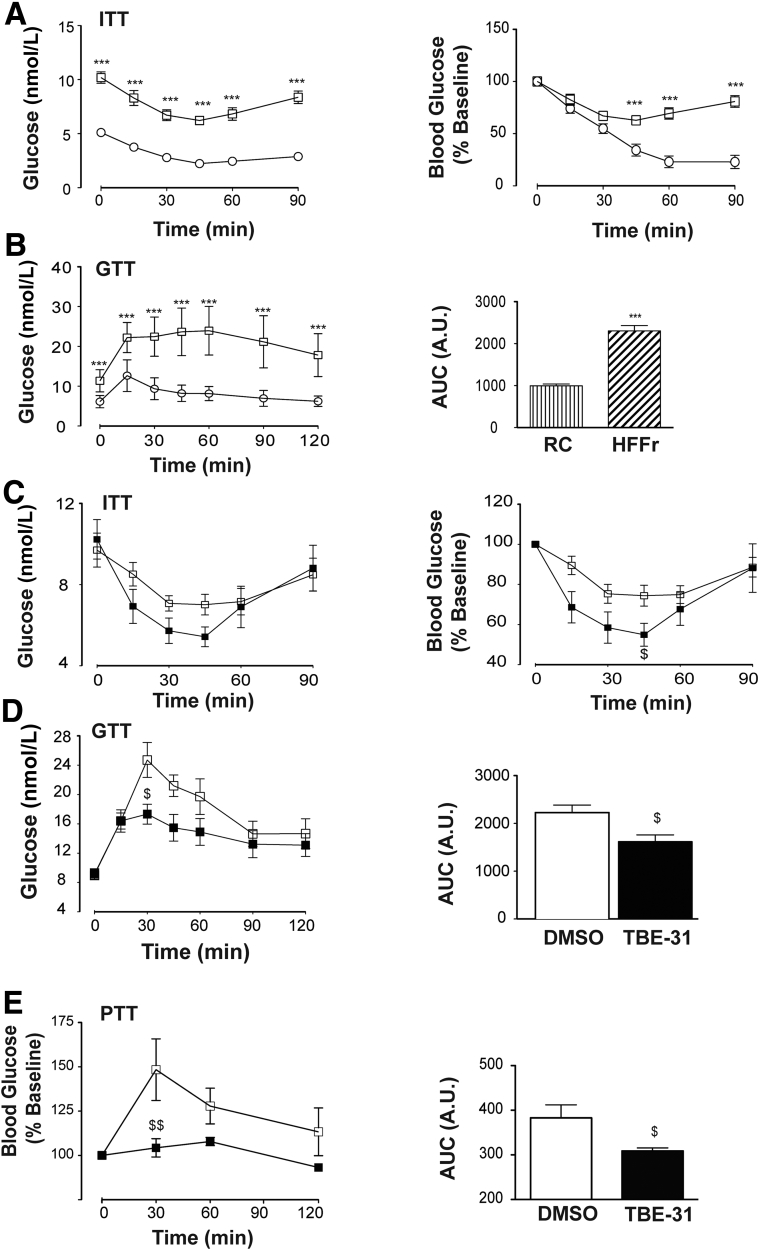
**TBE-31 improves insulin sensitivity in HFFr-fed *Nrf2***^***+/+***^**mice.** (*A*) Insulin sensitivity (ie, ITT) (and as % change in blood glucose) in *Nrf2*^*+/+*^ mice after 22 weeks RC- (*white circle*) or HF55Fr/HF30Fr (HFFr)- (*white square*) feeding. (*B*) Glucose tolerance (ie, GTT, with AUC) in *Nrf2*^*+/+*^ mice after 23 weeks RC (*white circle*) or HFFr (*white square*) feeding. (*C*) Insulin sensitivity (ITT) (and as % change in blood glucose) in *Nrf2*^*+/+*^ mice after 28 weeks HFFr diet, and 4 weeks DMSO (*white square*) or TBE-31 (*black square*). (*D*) Glucose tolerance (GTT, with AUC) in *Nrf2*^*+/+*^ mice after 29 weeks HFFr diet and 5 weeks DMSO (*white square*) or TBE-31 (*black square*). (*E*) Pyruvate tolerance (PTT) (and as % change in blood glucose, with AUC) in *Nrf2*^*+/+*^ mice after 29.5 weeks RC diet or HFFr diet, and 5 weeks DMSO (*white square*) or TBE-31 (*black square*). In *A* and *B*, n = 20–24 mice/group: in *C–E*, n = 6–8 mice/group. *White bars*, DMSO treated; *black bars*, TBE-31 treated. Data are means ± SEM: ^∗,$^*P* < .05; ^∗∗^*P* < .01; ^∗∗∗^*P* < .001. AUC, area under the curve.

**Figure 5:**
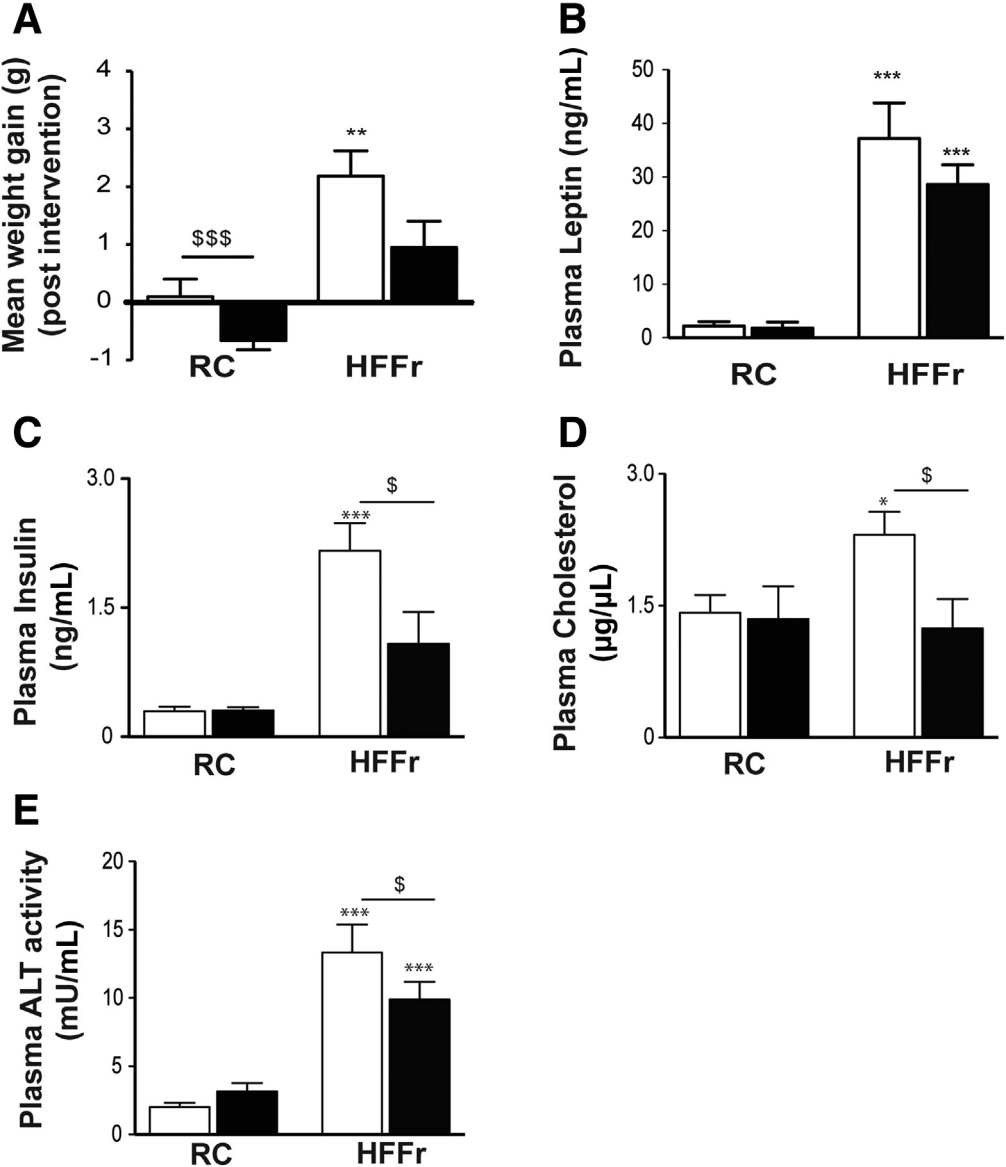
**TBE-31 attenuates weight gain and increases in plasma insulin, cholesterol and alanine aminotransferase in HFFr-fed *Nrf2***^***+/+***^**mice.** In Study 1, *Nrf2*^*+/+*^ mice were killed and blood collected after 30 weeks on either the RC or HF55Fr/HF30Fr (HFFr) diet. (*A*) Mean gain in body weight over the 6-week treatment period of mice on the RC or HFFr diets that were administered either TBE-31 or the DMSO control. (*B*) Plasma leptin levels in RC-fed and HFFr-fed mice treated with TBE-31 or DMSO control. (*C*) Plasma insulin levels in RC-fed and HFFr-fed mice treated with TBE-31 or DMSO control. (*D*) Plasma cholesterol levels in RC-fed and HFFr-fed mice treated with TBE-31 or DMSO control. (*E*) Plasma alanine aminotransferase activity in RC-fed and HFFr-fed mice treated with TBE-31 or DMSO control. *White bars*, DMSO treated; *black bars*, TBE-31 treated (n = 8–12 mice per group). Results are means ± SEM. Significant increases in results, relative to those in livers from RC-fed *Nrf2*^*+/+*^ mice, are indicated by: *P < .05, **P < .01, ***P < .001. Significant decreases in results as a consequence of treatment with TBE-31, relative to HFFr-fed *Nrf2*^*+/+*^ mice, are indicated by: ^$^*P* < .05, ^$$$^*P* < .001. ALT, alanine aminotransferase.

ITT and GTT analyses showed the 20 obese *Nrf2*^*+/+*^ HF55Fr/HF30Fr-fed mice exhibited impaired glucose clearance and were less responsive to insulin (measured at 22–23 weeks) than the RC-fed mice (Figure 4*A* and *B*). Following 9 weeks on the standard HF30Fr diet (ie, starting at the beginning of Week 25 of the HF55Fr/HF30Fr regimen), the obese *Nrf2*^*+/+*^ mice were randomly assigned to 2 groups that were either treated with TBE-31 or DMSO vehicle control, for 6 weeks, while continuing to be fed the HF30Fr diet. A second ITT and GTT, performed in Weeks 4 and 5, respectively, after TBE-31 administration commenced, showed that TBE-31-treated mice cleared glucose more effectively than vehicle control HF55Fr/HF30Fr-fed mice (Figure 4*C* and *D*). PTT also revealed that TBE-31 suppressed hepatic gluconeogenesis (Figure 4*E*). The improvement in glucose homeostasis in HF55Fr/HF30Fr-fed mice was associated with a decrease in weight gain (Figure 5*A*), but because this was not accompanied by a significant decrease in plasma leptin (Figure 5*B*) it is unlikely to be associated with differences in hunger. Interestingly, TBE-31 treatment decreased fasted plasma insulin and cholesterol levels, and plasma alanine aminotransferase activity, in HF55Fr/HF30Fr-fed mice (Figure 5*C*–*E*).

**Figure 6:**
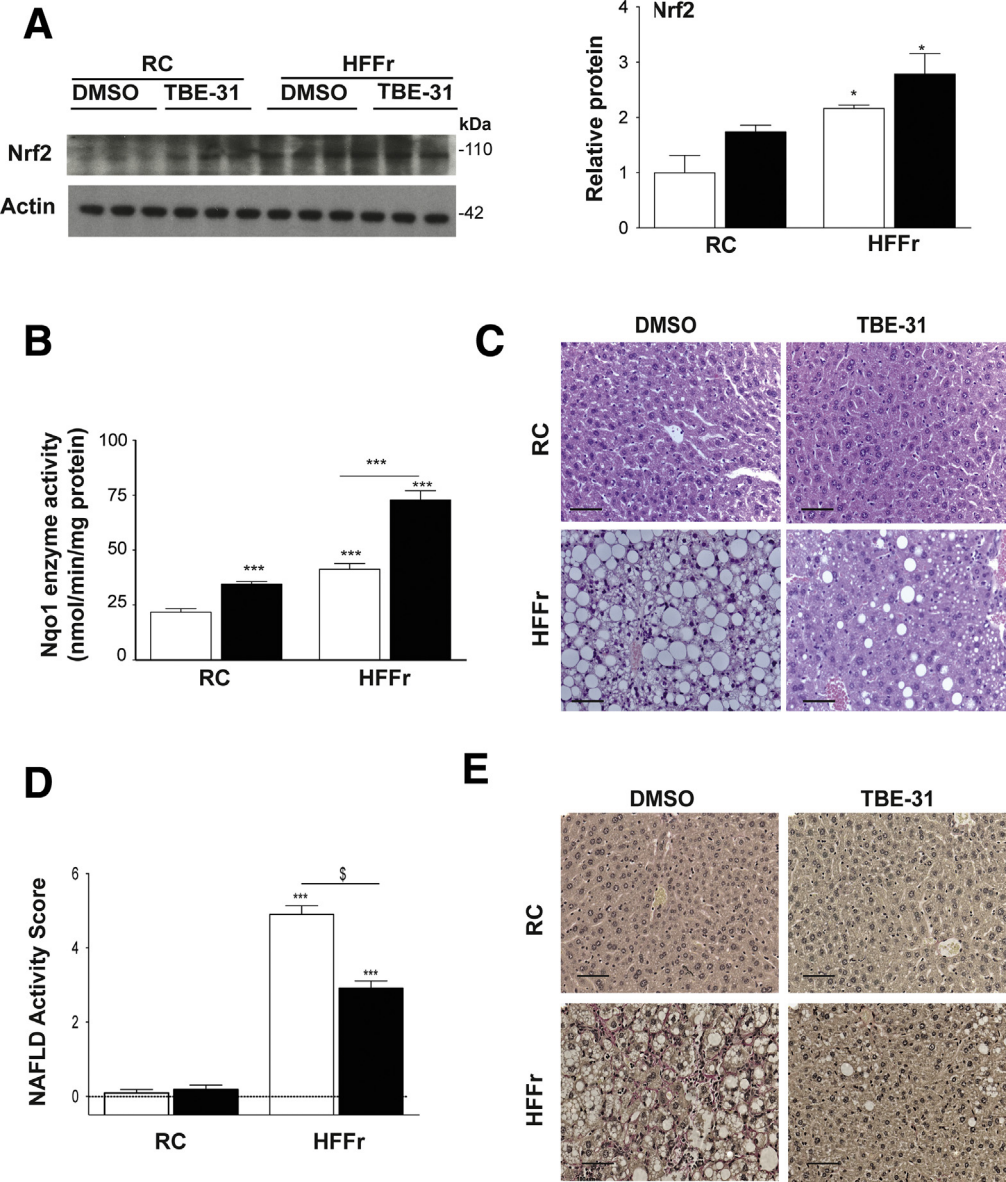
**TBE-31 treatment increases hepatic Nrf2 activity and improves liver histology in HFFr-fed *Nrf2***^***+/+***^**mice.** On completion of the Study 1 protocol, *Nrf2*^*+/+*^ mice were killed and livers removed. (*A*) A representative immunoblot for Nrf2 protein in liver extracts from RC-fed or HFFr-fed mice treated with DMSO or TBE-31 (*left side*), with densitometric scans of blots (*right side*) (n = 6 biologic replicates). (*B*) Nqo1 catalytic activity in hepatic extracts from RC-fed and HFFr-fed mice (n = 8–12 mice per group). (*C*) Representative images for H&E staining of liver sections from RC- and HFFr-fed *Nrf2*^*+/+*^ mice treated with DMSO or TBE-31 (scale bars = 100 μm). (*D*) The NAFLD activity score^39^ was calculated (n = 8–12 mice per group): note, on the y-axis the score includes negative values because livers from RC-fed *Nrf2*^*+/+*^ mice gave NAFLD activity scores of essentially zero. (*E*) Representative images for van Gieson staining of liver sections from *Nrf2*^*+/+*^ mice after 30 weeks RC or HF55Fr/HF30Fr feeding, followed by 6 weeks DMSO or TBE-31 treatment (scale bars = 100 μm). *White bars*, DMSO treated; *black bars*, TBE-31 treated. Results are means ± SEM. Significant increases in Nrf2 protein, Nqo1 activity, or NAFLD activity score, relative to that in livers from RC-fed *Nrf2*^*+/+*^ mice, are indicated by: *P < .05; ***P < .001. Significant decreases in NAFLD activity score upon treatment with TBE-31, relative to HFFr-fed *Nrf2*^*+/+*^ mice, are indicated by: ^$^*P* < .05.

Once the mice were killed, we first examined whether the 6-week treatment with TBE-31 had resulted in activation of Nrf2. Immunoblotting of liver extracts revealed that the abundance of Nrf2 protein was increased approximately 2-fold by the HFFr diet, and although TBE-31 treatment did not produce a significant further increase in Nrf2 abundance a trend was nevertheless apparent (Figure 6*A*). Most importantly, examination of Nqo1 catalytic activity, an enzyme that is a prototypic marker of Nrf2 transactivation activity, revealed a 1.6-fold increase in livers of TBE-31-treated RC-fed mice and a 1.8-fold increase in TBE-31-treated HF55Fr/HF30Fr-fed mice, each relative to their respective DMSO-treated controls (Figure 6*B*). H&E staining revealed that livers of *Nrf2*^*+/+*^ mice fed the high-calorie diet for 30 weeks, and given DMSO vehicle over the final 6 weeks, exhibited marked steatosis, inflammation, and hepatocyte ballooning (Figure 6*C*), which combined to give an average NAS of 4.9 (Figure 6*D*). By comparison, the number of hepatic steatotic vesicles and extent of inflammation and ballooning was diminished in HF55Fr/HF30Fr-fed mice administered TBE-31 over the final 6 weeks, giving an average NAS of 2.9. Individual scores for fat, inflammation, fibrosis, and hepatocyte ballooning revealed that TBE-31 decreased substantially hepatocyte ballooning (*P* < .05) and fibrosis (*P* < .05) (Table 2). Van Gieson staining also revealed TBE-31 substantially diminished fibrosis in livers of HF55Fr/HF30Fr-fed mice (Figure 6*E*).

**Table 2:** TBE-31 Decreases Histological Features Associated With NASH and Cirrhosis in Livers of Mice Fed a HFFr Diet

Parameter	Value (n)	
	DMSO	TBE-31
NAS^a^ (maximum 8)		
RC	0.091 (11)	0.182 (11)
HFFr	4.901 (8)	2.917 (8)
Steatosis component of NAS^b^ (0–3)		
RC	0.0 (11)	0.0 (11)
HFFr	2.900 (8)	1.833 (8)
Inflammatory component of NAS^c^ (0–3)		
RC	0.091 (11)	0.182 (11)
HFFr	1.300 (8)	1.000 (8)
Ballooning component of NAS^d^ (0–2)		
RC	0.0 (11)	0.0 (11)
HFFr	0.700 (8)	0.0833 (8)
Fibrosis stage^e^ (0–3)		
RC	0.0 (11)	0.0 (11)
HFFr	0.727 (8)	0.167 (8)

a The NAS-based evaluation of severity of NAFLD was found to be significantly higher in livers of HFFr-fed mice than in livers of RC-fed animals (Kruskal-Wallis test; *P* < .0001). Livers of mice fed the HFFr diet and treated with DMSO vehicle control had significantly higher NAS than those of HFFr-fed mice treated with TBE-31 (Kruskal-Wallis H test; *P* < .05).

b Livers of mice fed the HFFr diet showed more steatosis than livers of their RC-fed counterparts (Kruskal-Wallis H test; *P* < .0001). No significant difference was observed in liver steatosis between DMSO- and TBE-31-treated mice fed on the same diet.

c The inflammatory component was significantly higher in livers of mice fed on the HFFr diet when compared with their RC-fed counterparts (Kruskal-Wallis H test; *P* < .0001). No significant difference in liver inflammation was observed between DMSO- and TBE-31-treated mice on the same diet.

d Liver ballooning was significantly higher in mice fed the HFFr diet when compared with their RC-fed counterparts (Kruskal-Wallis H test; *P* < .0001). Ballooning in livers of mice fed the HFFr diet and treated with DMSO was significantly higher than in livers of HFFr-fed mice treated with TBE-31 (Kruskal-Wallis H test; *P* < .05).

e Liver fibrosis (this is not included in the NAS calculation) was significantly higher in mice fed the HFFr diet when compared with their RC-fed counterparts (Kruskal-Wallis H test; *P* < .0001). Fibrosis in livers of mice fed the HFFr diet and treated with DMSO was significantly higher than in livers of HFFr-fed mice treated with TBE-31 (Kruskal-Wallis H test; *P* < .05).

### TBE-31 Attenuates Expression of Lipid Metabolism Genes in Livers of HF55Fr/HF30Fr-Fed Mice

**Figure 7:**
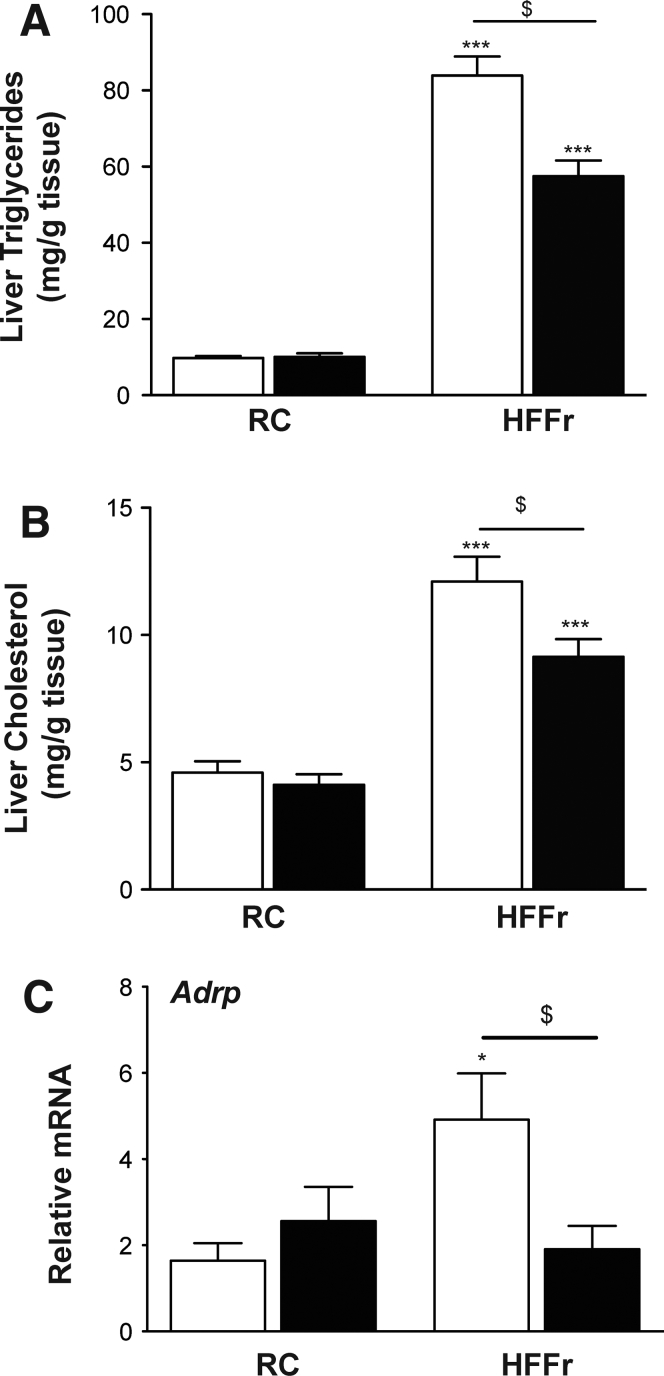
**TBE-31 decreases the abundance of triglycerides and cholesterol in the livers of HFFr-fed *Nrf2***^***+/+***^**mice.** Lipids and mRNA for Adrp were measured in livers from mice in Study 1. Triglyceride (*A*) and cholesterol (*B*) in livers from mice on the RC diet and mice on the HF55Fr/HF30Fr (HFFr) diet. (*C*) qRT-PCR for Adrp. *White bars*, DMSO-treated; *black bars*, TBE-31−treated (n = 8–12 mice per group). Results are means ± SEM. Significant increases in triglyceride or cholesterol levels, relative to those in livers from RC-fed *Nrf2*^*+/+*^ mice, are indicated by: *P < .05; ***P < .001. Significant decreases in hepatic triglyceride or cholesterol levels, or mRNA for Adrp, resulting from treatment with TBE-31, relative to HFFr-fed *Nrf2*^*+/+*^ mice, are indicated by: ^$^*P* < .05.

TBE-31 treatment decreased triglyceride and cholesterol levels in livers of *Nrf2*^*+/+*^ mice fed a HF55Fr/HF30Fr diet (Figure 7*A* and *B*). It also decreased expression of adipose differentiation-related protein (Adrp), a marker for the presence of lipid droplets,^41^ in livers of the high-calorie diet fed *Nrf2*^*+/+*^ mice (Figure 7*C*), which is consistent with the steatosis observed on H&E staining of liver sections.

**Figure 8:**
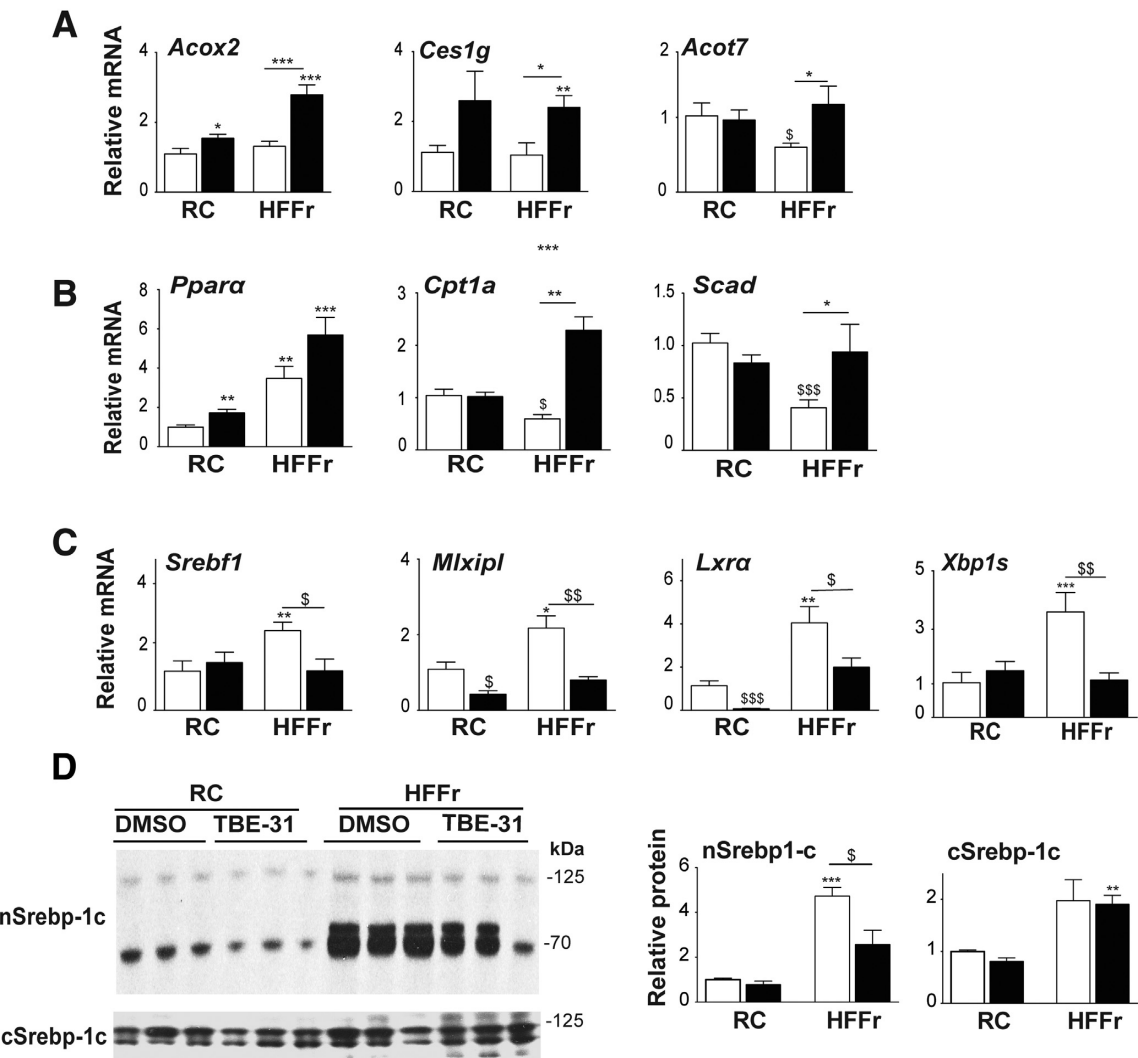
**TBE-31 stimulates lipid catabolism and suppresses lipogenic transcription factors.** On completion of Study 1, livers were removed from *Nrf2*^*+/+*^ mice and portions examined for expression of lipid-associated genes and protein analyses of the transcription factor Srebp-1c. (*A*) qRT-PCR for *Acox2*, *Ces1g*, and *Acot7*, (*B*) qRT-PCR for *PPARα, Cpt1a*, and *Scad*, and (*C*) qRT-PCR for *Srebf1*, *Mlxipl*, *Lxrα*, and *Xbp1s* (n = 8–12 mice per group). (*D*) A representative Srebp-1c immunoblot of cytoplasmic (cSrebp-1c) and nuclear (nSrebp-1c) protein (*left side*), with densitometric scans of blots (*right side*) (n = 6 biologic replicates). *White bars*, DMSO; *black bars*, TBE-31. Data are means ± SEM. Significant increases in gene expression or protein abundance, relative to that in livers from RC-fed *Nrf2*^*+/+*^ mice, are indicated by: *P < .05; **P < .01; ***P < .001. Significant decreases in gene expression or protein abundance, relative to that in livers from RC-fed *Nrf2*^*+/+*^ mice, are indicated by: ^$^*P* < .05; ^$$^*P* < .05.

Because Nrf2 positively controls lipid catabolism genes,^19^ we explored whether these were induced by TBE-31 treatment and found TBE-31 increased hepatic mRNA for acetyl-CoA oxidase 2 (Acox2), carboxylesterase 1g (Ces1g), and acetyl-CoA thioesterase 7 (Acot7) (Figure 8*A*). Changes in fatty acid oxidation genes were also observed, with the HF55Fr/HF30Fr diet increasing mRNA for peroxisome proliferator-activated receptor α (Pparα), and decreasing carnitine palmitoyltransferase 1a (Cpt1a) and short-chain acyl-CoA dehydrogenase (Scad), with TBE-31 intervention raising levels of all 3 of the mRNA species (Figure 8*B*).

We considered whether decreased hepatic steatosis in HF55Fr/HF30Fr-fed mice treated with TBE-31 might involve diminished *de novo* lipogenesis. Among transcription factors that control lipid metabolism, mRNAs for Srebp-1c and Chrebp (encoded by *Srebf1* and *Mlxipl*, respectively) and the liver X receptor α (Lxrα, also called Nr1h3) and Xbp1s, were increased by the HF55Fr/HF30Fr diet, and treatment of mice fed this high-calorie diet with TBE-31 decreased their expression (Figure 8*C*). We also found that mRNA for the lipogenic transcription factors Pparγ and C/ebpα were induced approximately 4.0-fold by the HF55Fr/HF30Fr diet but this was not altered significantly by treatment with TBE-31 (data not shown). By contrast, mRNA for C/ebpβ was not significantly changed by either the HF5Fr/HF30Fr diet or by TBE-31 (data not shown). Importantly, immunoblotting showed higher levels of Srebp-1c protein in nuclear fractions from livers of HF55Fr/HF30Fr-fed mice than in their RC-fed counterparts, and this was decreased by TBE-31 treatment (Figure 8*D*). Because Srebp-1c and Xbp1s have been linked to the UPR,^12^, ^42^ their apparently coordinated decrease in expression following treatment with TBE-31 suggests that ER stress might be attenuated (see below).

Because LXRα is regulated independently of ER stress,^43^ we explored whether levels of mRNA for the farnesoid X receptor (Fxr) and small heterodimer partner (Shp) were altered by the diet, or by TBE-31 treatment, because they have both been implicated in repression of *Lxrα*.^44^ Quantitative reverse transcriptase PCR (qRT-PCR) revealed that neither Fxr nor Shp mRNA levels were increased by the HF55Fr/HF30Fr diet, and neither was induced by TBE-31 treatment (data not shown). Similar analysis of mRNA for retinoid X receptor alpha (Rxrα), which forms a heterodimer with Lxrα, revealed that its expression was also not affected by either diet or TBE-31 (data not shown).

**Figure 9:**
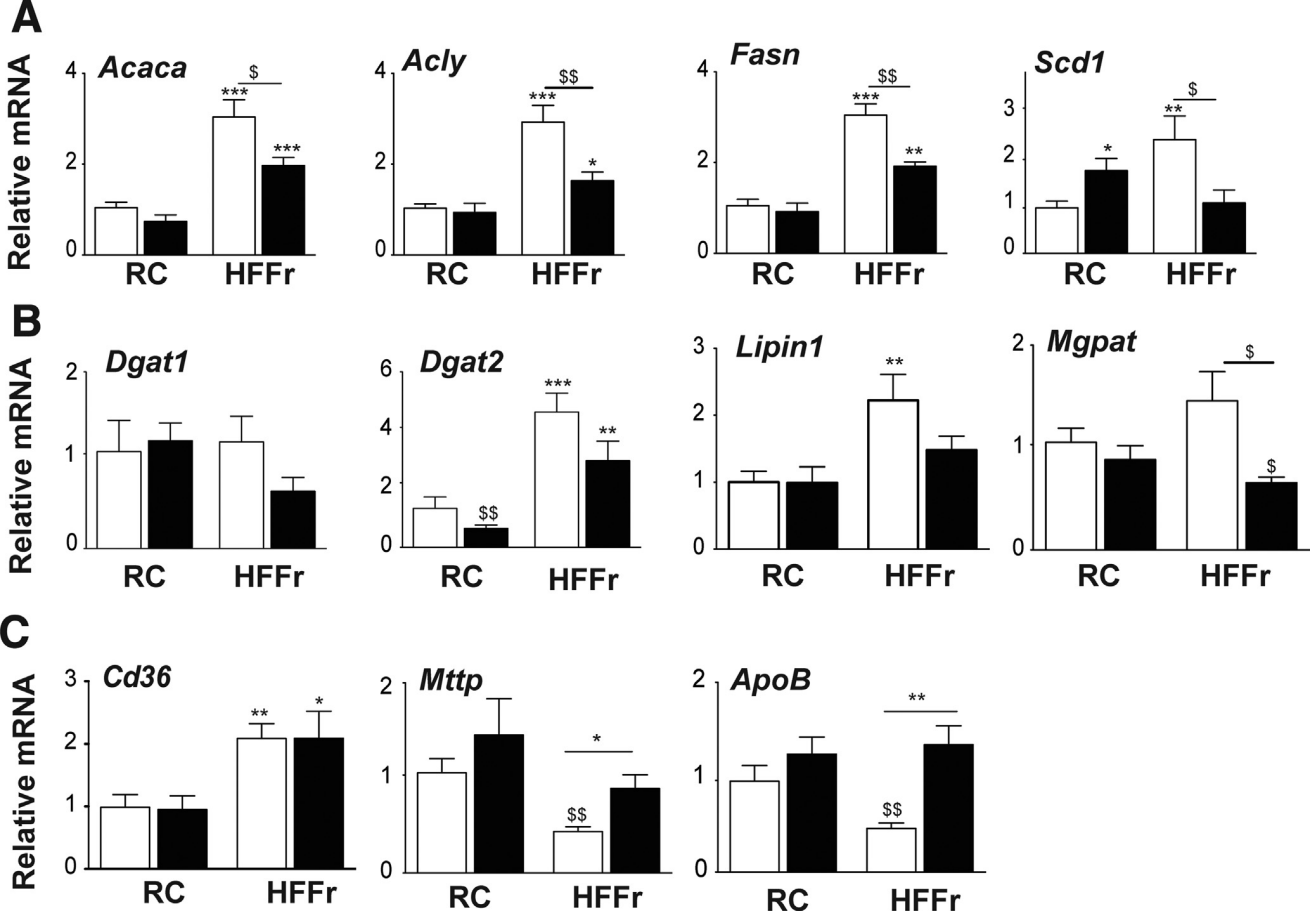
**TBE-31 suppresses expression of genes for lipid synthesis enzymes but increases expression of lipid exporters.** Expression of lipid synthesis enzymes and lipid transporters were examined in livers of mice from *Study-1*. (*A*) qRT-PCR for *Acaca*, *Acly*, *Fasn*, and *Scd1*. (*B*) qRT-PCR for *Dgat1*, *Dgat2*, *Lipin1*, and *Mgpat*. (*C*) qRT-PCR for *Cd36*, *Mttp*, and *ApoB*. *White bars*, DMSO; *black bars*, TBE-31 (8–12 mice per group). Data are means ± SEM. Significant changes are indicated: ^∗,$^*P* < .05; ^∗∗,$$^*P* < .01; ^∗∗∗^*P* < .001.

Next, we examined whether downregulation of the lipogenic transcription factors noted previously resulted in decreased expression of lipid-synthesis enzymes. This revealed the HF55Fr/HF30Fr diet increased mRNA for the fatty acid synthesis enzymes acetyl-CoA carboxylase alpha (Acaca), ATP citrate lyase (Acly), fatty acid synthase (Fasn), and stearoyl-CoA desaturase-1 (Scd1), with TBE-31 diminishing substantially induction of these genes by the diet (Figure 9*A*). The HF55Fr/HF30Fr diet also stimulated modest increases in mRNA for the triglyceride synthesis enzymes diacylglycerol acyltransferase-2 (Dgat2), lipin-1, and mitochondrial glycerol-3-phosphate acetyltransferase (Mgpat), with the induction of each decreased by TBE-31, although Dgat1 was unaffected (Figure 9*B*). Thus TBE-31 suppresses liver steatosis, at least in part, by attenuating dietary stimulation of expression and activation of Srepb-1c, Chrebp, Lxrα, and Xbp1s, which consequently lowers expression of lipogenic enzymes.

To assess whether a decrease in lipid import or an increase in lipid export contributes to the reduction in hepatic steatosis affected by TBE-31, we measured mRNA for cluster of differentiation 36 (Cd36), which contributes to lipid import, and microsomal triglyceride transfer protein (Mttp) and apolipoprotein B (ApoB), which contribute to lipid export. This revealed that the HF55Fr/HF30Fr diet increased Cd36 mRNA levels but that TBE-31 did not alter it (Figure 9*C*). By contrast, although the HF55Fr/HF30Fr diet suppressed Mttp and ApoB mRNA levels, TBE-31 increased their abundance (Figure 9*C*). Collectively, these results suggest that TBE-31 probably influences lipid transport to just a limited degree.

### Treatment With TBE-31 Suppresses Endoplasmic Reticulum Stress in Livers of HF55Fr/HF30Fr-Fed Mice

**Figure 10:**
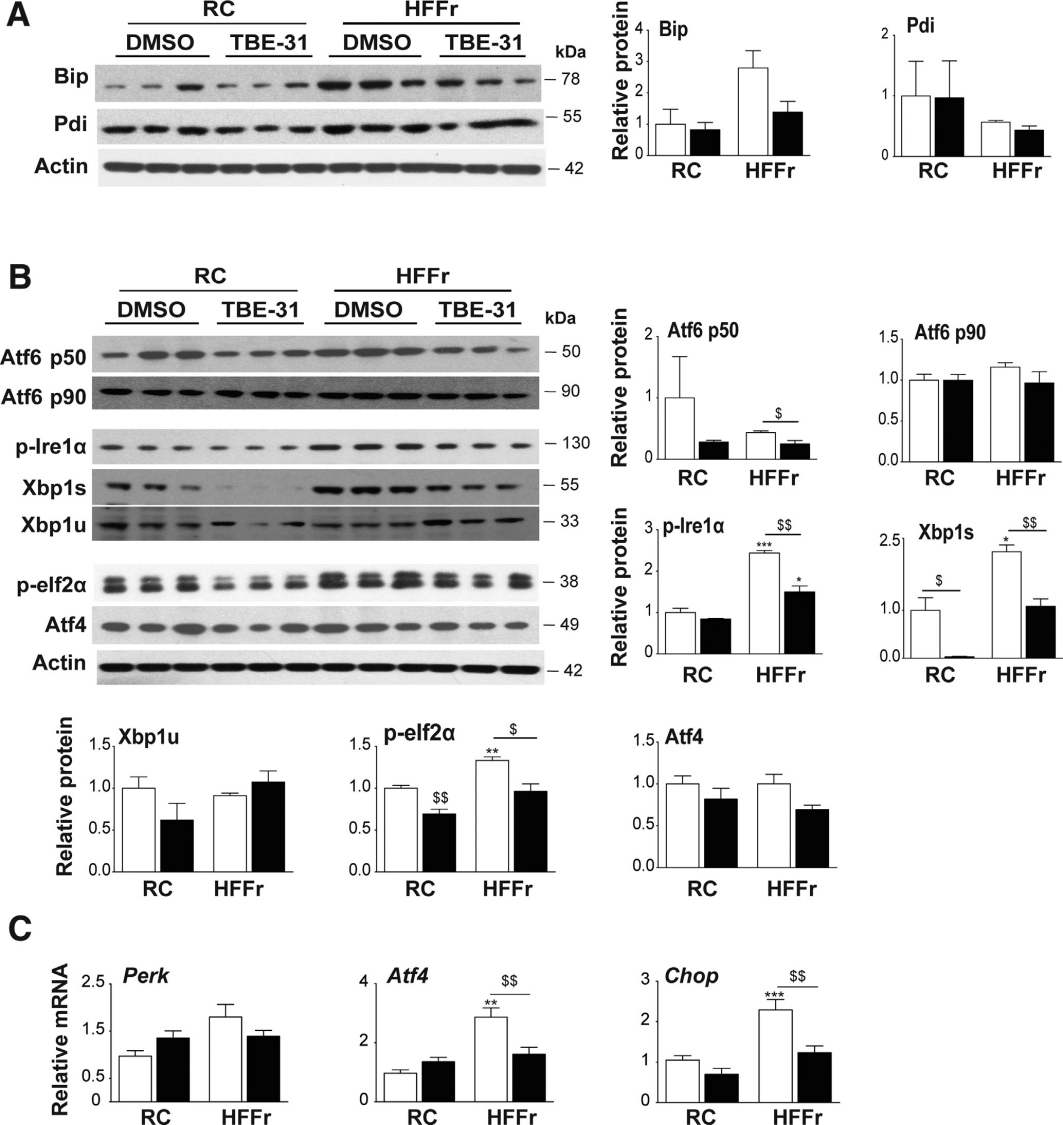
**TBE-31 suppresses ER stress in livers of *Nrf2***^***+/+***^**mice fed a HFFr diet.** Livers from *Nrf2*^*+/+*^ mice in Study 1 were examined for changes in proteins and genes engaged in the UPR. (*A*) Representative immunoblots of Bip and Pdi (with actin as loading control) in hepatic extracts from RC-fed and HF55Fr/HF30Fr (HFFr)-fed mice treated with DMSO or TBE-31, along with densitometric scans of blots (n = 6 biologic replicates). (*B*) Representative immunoblots of Atf6 p50 and p90, p-Ire1α, Xbp1s and Xbp1u, p-eIf2α, and Atf4 (with actin as loading control) along with densitometric scans of blots as indicated (n = 6 biologic replicates). (*C*) qRT-PCR for *Perk*, *Atf4*, and *Chop* (n = 8–12 mice per group). In all cases, *white bars* represent DMSO and *black bars* represent TBE-31. Data are means ± SEM. Significant changes are indicated: ^∗,$^*P* < .05; ^∗∗,$$^*P* < .01; ^∗∗∗^*P* < .001.

Because TBE-31 decreased expression of *Srebf1* and *Xbp1s*, we next explored whether TBE-31 antagonizes steatosis by suppressing ER stress. Initially, we examined the abundance of Bip and Pdi because their expression is increased on activation of the UPR.^45^ Immunoblotting showed the HF55Fr/HF30Fr diet increased Bip levels in liver of *Nrf2*^*+/+*^ mice, and that treatment with TBE-31 attenuated the increase (Figure 10*A*); this was not, however, apparent for Pdi. Further immunoblotting indicated that all 3 arms of the UPR were activated in livers of HF55Fr/HF30Fr-fed mice when compared with RC-fed mice, and the abundance of these markers was decreased by TBE-31 treatment: evidence for activation of Ire1α was deduced from increases in phospho-Ire1α, for activation of Atf6 by increases in Atf6-p50, and for activation of Perk by increases in phospho-eIf2α (Figure 10*B*). Hepatic levels of mRNA for Atf4 (downstream of Perk) and C/ebp homologous protein (Chop) (downstream of Perk) were also increased by the high-calorie diet and attenuated by TBE-31 (Figure 10*C*). Together, these data indicate that consumption of the high-calorie diet stimulates ER stress and this can be mitigated by TBE-31. Thus, alleviation of ER stress by TBE-31 likely contributes to suppression of hepatic steatosis by blunting induction of *Srebf1* and *Xbp1s*.

### TBE-31 Treatment of HF55Fr/HF30Fr-Fed Mice Decreases Hepatic Inflammation, Apoptosis and Fibrosis

**Figure 11:**
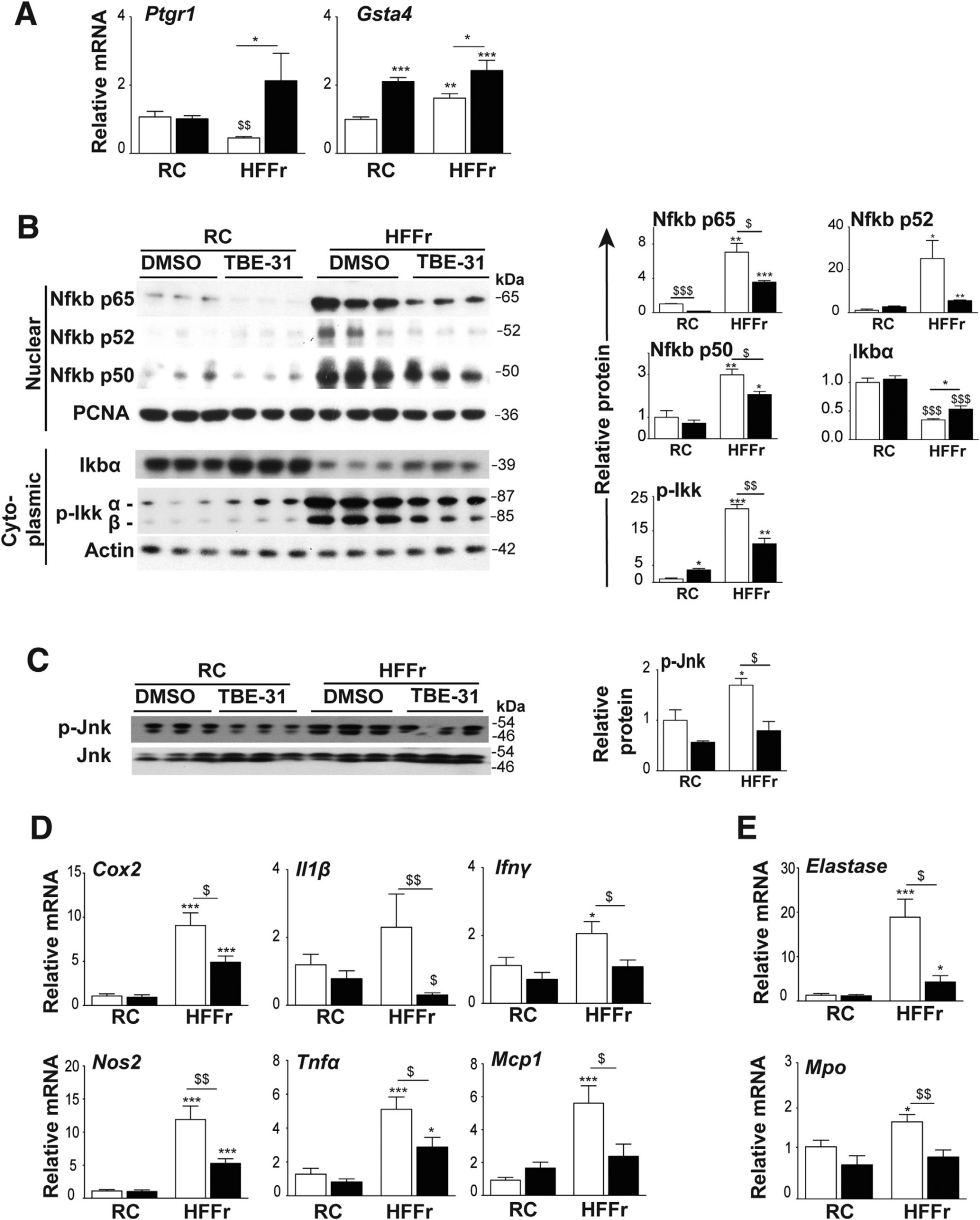
**TBE-31 suppresses hepatic inflammation in HFFr-fed *Nrf2***^***+/+***^**mice.** Expression of anti-inflammatory genes and abundance of proinflammatory proteins and proinflammatory genes was examined in livers of *Nrf2*^*+/+*^ mice from Study 1. (*A*) qRT-PCR for *Ptgr1* and *Gsta4* (8–12 mice per group). (*B*) Representative immunoblots of Nfkb p65, p52, and p50 nuclear fraction proteins, with proliferating cell nuclear antigen as loading control, and cytoplasmic Ikbα and p-Ikkα/β with actin as loading control; densitometric scans of blots are shown alongside (n = 6 biologic replicates). (*C*) Representative immunoblots of p-Jnk and Jnk, with densitometric scans shown adjacent (n = 6 biologic replicates). (*D*) qRT-PCR for *Cox2*, *Il1β*, *Ifnγ*, *Nos2*, *Tnfα*, and *Mcp1* (n = 8–12 per group). (*E*) qRT-PCR for *Elastase* and *Mpo* (8–12 mice per group). In all cases, *white bars* represent DMSO and *black bars* represent TBE-31. Data are means ± SEM. Significant differences are denoted: ^∗,$^*P* < .05; ^∗∗,$$^*P* < .01; ^∗∗∗,$$$^*P* < .001.

Nrf2-target genes include those encoding enzymes that metabolize proinflammatory lipid-derived reactive aldehydes, such as prostaglandin reductase-1 (Ptgr1) and glutathione *S-*transferase Alpha-4 (Gsta4).^19^ HF55Fr/HF30Fr suppressed mRNA for Ptgr1, which was recovered by TBE-31 treatment (Figure 11*A*). By contrast, HF55Fr/HF30Fr increased mRNA for Gsta4, with TBE-31 inducing it further. Messenger RNA for other potential Nrf2-regulated aldehyde-metabolizing enzymes were not induced by TBE-31 (data not shown), suggesting they do not contribute to suppression of hepatic inflammation.

Immunoblotting revealed marked increases in nuclear levels of Nfkb p65, p52, and p50 proteins in livers from HF55Fr/HF30Fr-fed mice, which were attenuated by TBE-31 (Figure 11*B*). Remarkably, the dramatic increases in hepatic nuclear Nfkb p65, p52, and p50 protein in mice fed the high-calorie diet, and the attenuation by TBE-31 treatment, were accompanied by correspondingly large decreases in Ikbα protein and large increases in Ikkα/β phosphorylation (Figure 11*B*). The HF55Fr/HF30Fr diet also increased Jnk phosphorylation, which was suppressed by TBE-31 (Figure 11*C*). Taken together with the data in Figure 10, these results suggest that ER stress in the livers of HF55Fr/HF30Fr-fed mice may stimulate Nfkb-directed transcription by decreasing translation of Ikbα (downstream of Perk) and by increasing Ikk activity (downstream of Ire1α). Moreover, the ability of TBE-31 to suppress ER stress in livers of HF55Fr/HF30Fr-fed mice seems to be reflected in a diminution of diet-stimulated increases in nuclear Nfkb protein and Ikk phosphorylation, coupled with enhanced levels of Ikb protein.

Consistent with these results for Nfkb, we found the high-calorie diet increased hepatic mRNA for the Nfkb-targets cyclooxygenase-2 (Cox2) and nitric oxide synthase-2 (Nos2), and to a lesser degree mRNA for Il-1β, interferon-γ (Ifnγ), monocyte chemotactic protein-1 (Mcp-1), and tumor necrosis factor-α (Tnfα) (Figure 11*D*). Importantly, in livers of HF55Fr/HF30Fr-fed mice, TBE-31 decreased mRNAs for Cox2, Nos2, Il-1β, Ifnγ, Tnfα, and Mcp-1. Expression of the neutrophil markers elastase and myeloperoxidase (Mpo) was higher in livers of HF55Fr/HF30Fr-fed mice than RC-fed mice, and this was greatly attenuated by TBE-31 (Figure 11*E*). Collectively, these data suggest TBE-31 decreases activation of Ikk/Nfkb and Jnk inflammatory pathways in livers of mice caused by chronic consumption of the HF55Fr/HF30Fr diet, and diminishes recruitment of neutrophils into the liver.

**Figure 12:**
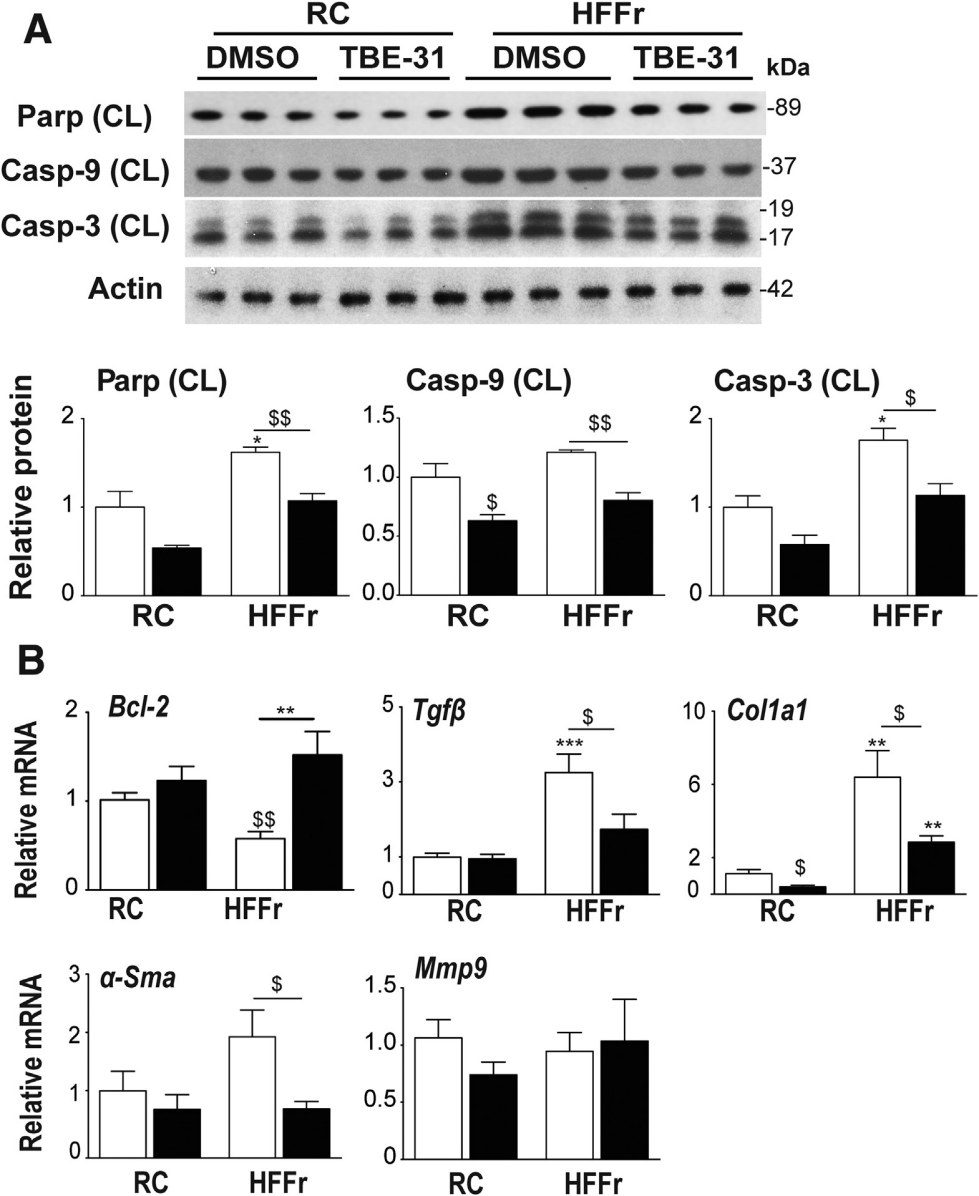
**TBE-31 suppresses hepatic apoptosis and fibrosis in HFFr-fed *Nrf2***^***+/+***^**mice.** The expression of apoptosis-associated proteins and fibrosis-associated genes was examined in livers of *Nrf2*^*+/+*^ mice from Study 1. (*A*) Representative immunoblots of cleaved (CL) Parp, caspase-9 (Casp-9), and caspase-3 (Casp-3), and actin as a loading control, along with densitometric scans (n = 6 biologic replicates). (*B*) qRT-PCR for *Bcl-2*, *Tgfβ*, *Col1a1*, *α-Sma*, and *Mmp9* (8–12 mice per group). In all cases, *white bars* represent DMSO and *black bars* represent TBE-31. Data are means ± SEM. Significant differences are signified: ^∗,$^*P* < .05; ^∗∗,$$^*P* < .01; ^∗∗∗^*P* < .001.

Cleavage of Parp, caspase-3 (Casp-3), and caspase-9 (Casp-9), which is indicative of apoptosis, was modestly increased in livers of HF55Fr/HF30Fr-fed *Nrf2*^*+/+*^ mice, and this was suppressed by TBE-31 (Figure 12A). Conversely, mRNA for the apoptosis suppressor Bcl-2 was increased by TBE-31 (Figure 12*B*). We next examined fibrosis. qRT-PCR revealed the HF55Fr/HF30Fr diet increased mRNA for transforming growth factor beta-1 (Tgfβ), a marker of hepatic stellate cell activation, which was diminished by TBE-31, as were the mRNAs for the fibrosis markers collagen, type I, alpha-1 (Col1a1) and alpha smooth muscle actin (α-Sma), but not that for matrix metallopeptidase 9 (Mmp9) (Figure 12*B*). These results suggest that treatment of HF55Fr/HF30Fr-fed *Nrf2*^*+/+*^ mice with TBE-31 diminishes apoptosis, and also fibrosis.

### TBE-31 Treatment of Mice Fed an HF55Fr/HF30Fr Diet Decreases Oxidative Stress

**Figure 13:**
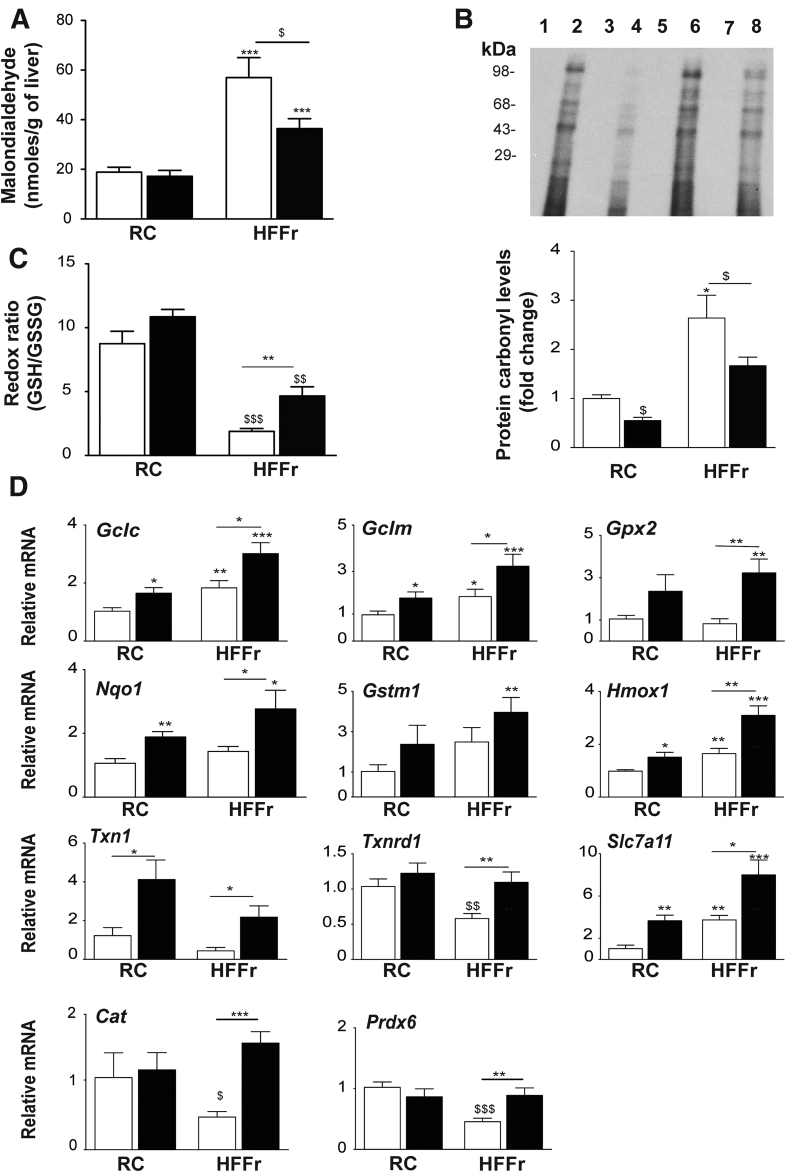
**TBE-31 suppresses oxidative stress in HFFr-fed *Nrf2***^***+/+***^**mice.** The abundance of oxidative stress-associated biomarkers and expression of antioxidant Nrf2-target genes examined in livers from Study 1. (*A*) Malondialdehyde levels. (*B*) Oxidized protein levels shown as a representative Oxyblot, with densitometric quantification below. Lanes 1, 3, 5, and 7 negative controls; 2, RC-fed DMSO; 4, RC-fed TBE-31; 6, HFFr-fed DMSO; 8, HFFr-fed TBE-31. (*C*) Ratio of GSH to GSSG. (*D*) qRT-PCR for *Gclc*, *Gclm*, *Gpx2*, *Nqo1*, *Gstm1*, *Hmox1*, *Txn1*, *Txnrd1*, *Slc7a11*, *Catalase* (*Cat*), and *Prdx6*. In all cases, *white bars* represent DMSO and *black bars* represent TBE-31. In *A*, *C*, and *D*, n = 8–12. In *B*, n = 6. Data are means ± SEM. Significant differences are represented as: ^∗,$^*P* < .05; ^∗∗,$$^*P* < .01; ^∗∗∗,$$$^*P* < .001.

Consistent with the notion that NASH is accompanied by oxidative stress, increases in malondialdehyde and oxidized protein were observed in livers of HF55Fr/HF30Fr-fed mice, and these increases were attenuated by TBE-31 treatment (Figure 13*A* and *B*). Moreover, the high-calorie diet decreased hepatic levels of reduced glutathione (GSH) relative to that of oxidized glutathione (GSSG), which is indicative of a more oxidized intracellular environment, with the relative abundance of GSH increased by TBE-31 treatment (Figure 13*C*). We also assessed whether the HF55Fr/HF30 diet and TBE-31 treatment affected expression of Nrf2-target genes that contribute to antioxidant defenses. This revealed that the high-calorie diet increased hepatic mRNA for glutamate-cysteine ligase catalytic (Gclc) and modifier (Gclm) subunits, heme oxygenase-1 (Hmox1) and solute carrier family 7 member 11 (Slc7a11), but not Nqo1, glutathione S-transferase Mu-1 (Gstm1), glutathione peroxidase-2 (Gpx2), thioredoxin-1 (Txn1) or thioredoxin reductase-1 (Txnrd1), catalase (Cat), or peroxiredoxin 6 (Prdx6). However, the abundance of all these mRNA species was increased by TBE-31 (Figure 13*C*), findings that suggest TBE-31 helps restore normal redox homeostasis in livers of HF55Fr/HF30Fr-fed mice by inducing Nrf2-regulated antioxidant genes.

### TBE-31 Requires Nrf2 to Increase Insulin Sensitivity and Ameliorate Adverse Liver Histology in HF30Fr-Fed Mice

**Figure 14:**
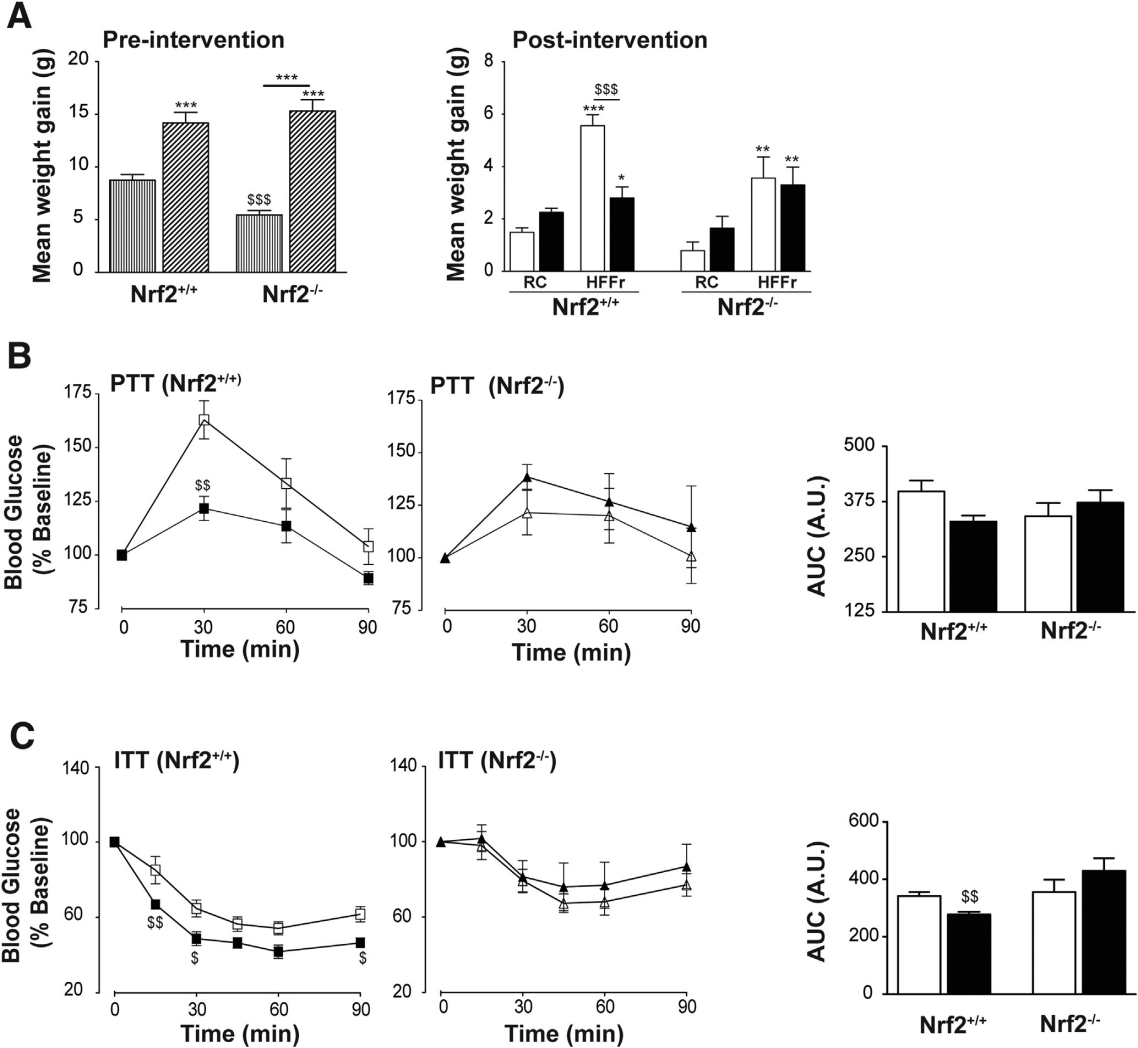
**TBE-31 fails to improve insulin sensitivity in HFFr-fed *Nrf2***^***-/-***^**mice.** During Study 2, physiological end-points and glucose homeostasis were examined in wild-type and Nrf2-null mice fed an RC or HF30Fr (HFFr) diet (n = 6–8 mice per group). (*A*) Body weight gain of mice up until intervention at end of Week 10 (left, *vertical striped bars*, RC; *diagonal striped bars*, HFFr) and following (Weeks 11–16) of treatment (*right*). (*B*) Glucose production (pyruvate tolerance) with AUC in *Nrf2*^*+/+*^ (*squares*) and *Nrf2*^*-/-*^ (*triangles*) mice after 10 weeks HF30Fr diet, followed by 5 weeks treatment with DMSO (*white squares and triangles*) or TBE-31 (*black squares and triangles*). (*C*) Insulin sensitivity (% change in blood glucose) in *Nrf2*^*+/+*^ (*squares*) and *Nrf2*^*-/-*^ (*triangles*) mice after 10 weeks HF30Fr diet followed by 4 weeks with DMSO (*white squares and triangles*) or TBE-31 (*black squares and triangles*). *White bars*, DMSO; *black bars*, TBE-31. Data are means ± SEM. Significant changes: ^∗^*P* < .05; ^∗∗,$$^*P* < .01; ^∗∗∗,$$$^*P* < .001. *D–F*, Scale bars = 100 μm. AUC, area under the curve,

Study 2 was instigated to determine whether Nrf2 is essential for TBE-31 to improve glucose use in obese mice and to mitigate NASH. When placed on the standard HF30Fr diet, *Nrf2*^*+/+*^ mice rapidly gained weight before treatment with TBE-31 or DMSO (Figure 14*A*); the *Nrf2*^*-/-*^ mice also gained substantial weight when placed on the HF30Fr diet, relative to RC-fed *Nrf2*^*-/-*^ mice, but a well-recognized feature of the mutant mice is that they are thinner than wild-type mice. Subsequently, however, when HF30Fr-fed *Nrf2*^*+/+*^ mice were treated with TBE-31 they gained less weight than *Nrf2*^*+/+*^ mice treated with DMSO. This diminution of weight gain in HF30Fr-fed wild-type mice on treatment with TBE-31 was not observed in HF30Fr-fed Nrf2-null mice. In both wild-type and Nrf2-null mice, the weight gain caused by the HF30Fr diet was accompanied by increases in plasma leptin levels, but these increases in leptin were not attenuated by TBE-31 treatment (data not shown). Both wild-type and Nrf2-null mice that had been placed on the HF30Fr diet for 9 weeks exhibited impaired PTT and ITT. Although treatment of HF30Fr-fed wild-type mice for 5 weeks with TBE-31 reduced glucose production (determined by PTT) (Figure 14*B*) and improved insulin sensitivity (determined by ITT) (Figure 14*C*), treatment of HF30Fr-fed *Nrf2*^*-/-*^ mice had substantially diminished beneficial effects.

**Figure 15:**
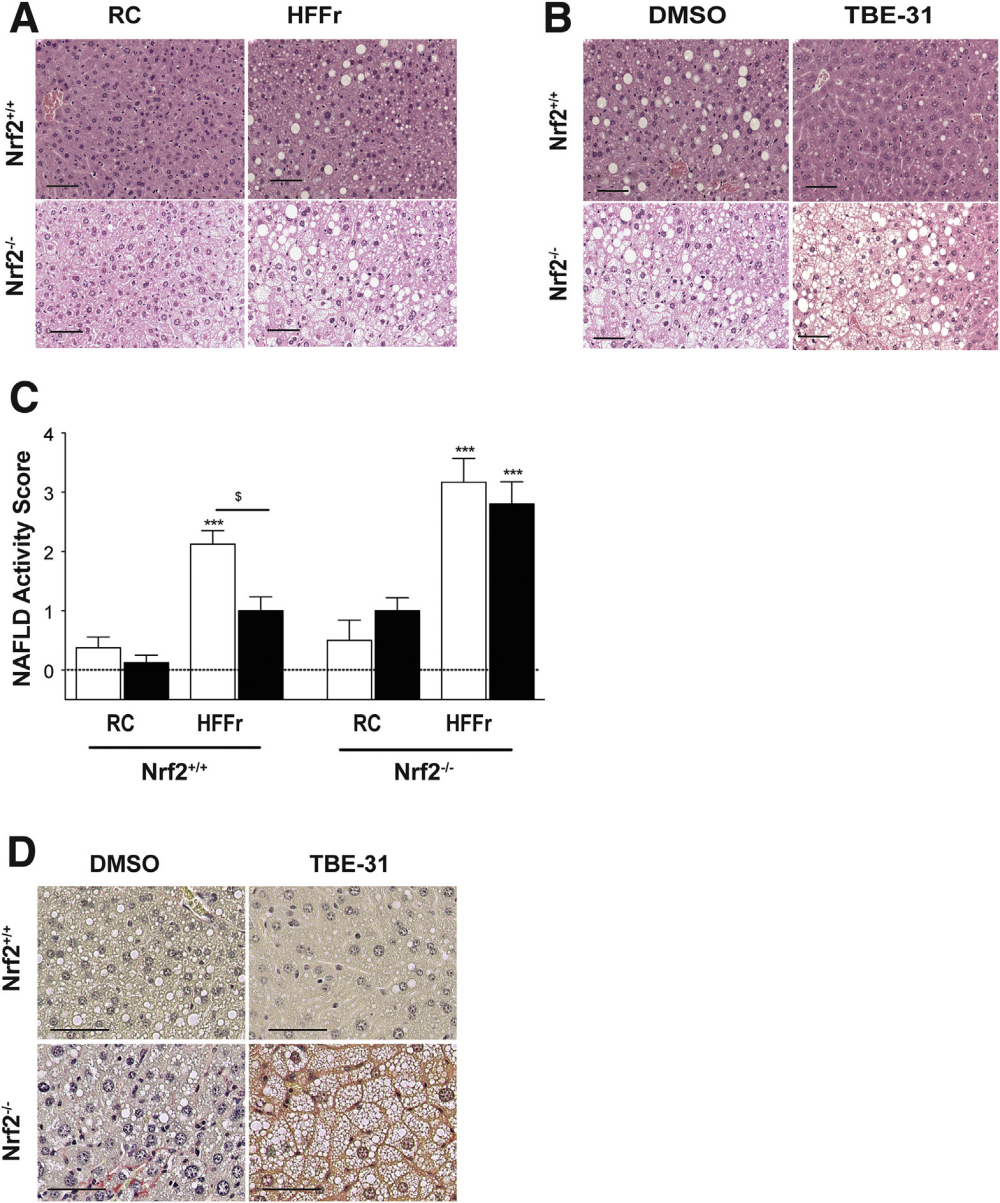
**TBE-31 does not improve NASH histology in livers of HFFr-fed *Nrf2***^***-/-***^**mice.** After sacrifice, livers from *Nrf2*^*+/+*^ and *Nrf2*^*-/-*^ mice in Study 2 were removed and fixed in formalin (n = 6–8 mice per group). (*A*) Representative images for H&E staining of mouse liver sections after 16 weeks RC- or HFFr-feeding, including treatment with DMSO during Weeks 11–16 (scale bars = 100 µm). (*B*) Representative images for H&E staining of liver sections from *Nrf2*^*+/+*^ and *Nrf2*^*-/-*^ mice after 16 weeks RC- or HFFr-feeding including treatment with DMSO or TBE-31 during Weeks 11–16 (scale bars = 100 µm). (*C*) The extent of disease was assessed using the NAFLD activity score method.^39^ (*D*) Representative images for van Gieson staining of liver sections from *Nrf2*^*+/+*^ and *Nrf2*^*-/-*^ mice after 16 weeks of HFFr-feeding and treatment with DMSO or TBE-31. *White bars*, DMSO-treated; *black bars*, TBE-31 treated (6–8 mice per group). Results are means ± SEM. Significant increases in NAFLD activity score, relative to that in livers from RC-fed DMSO-treated *Nrf2*^*+/+*^ mice, are indicated by: ***P < .001. The significant decrease in NAFLD activity score resulting from treatment with TBE-31, relative to HFFr-fed DMSO-treated *Nrf2*^*+/+*^ mice, is denoted by: ^$^*P* < .05.

H&E staining of liver sections revealed that consumption of the HF30Fr diet for 16 weeks stimulated greater steatosis and inflammation in *Nrf2*^*-/-*^ mice than in their wild-type counterparts (Figure 15*A*), and that TBE-31 treatment of mice on the HF30Fr diet decreased steatosis and inflammation in wild-type livers, but not in *Nrf2*^*-/-*^ livers (Figure 15B). Histologic evaluation of steatosis, inflammation, and hepatocyte ballooning showed that the average NAS in livers of *Nrf2*^*+/+*^ mice was reduced from 2.2 to 1.0 by TBE-31 (Figure 15*C*, Table 3). By contrast, the average NAS in livers of *Nrf2*^*-/-*^ mice, estimated to be 3.1, was not reduced by TBE-31. Examination of the H&E-stained sections revealed significantly greater fibrosis in Nrf2-null livers than wild-type livers. Similarly, van Gieson staining revealed fibrosis only in livers of *Nrf2*^*-/-*^ mice fed the HF30Fr diet (Figure 15*D*).

**Table 3:** Histological Examination Reveals That TBE-31 Does not Decrease the Severity of Liver Fibrosis in Nrf2-null Mice Fed a HFFr Diet

Parameter	Value (n)			
	*Nrf2*^*+/+*^ mice		*Nrf2*^*-/-*^ mice	
	DMSO	TBE-31	DMSO	TBE-31
NAS^a^ (maximum 8)				
RC	0.375 (8)	0.125 (8)	0.5 (6)	1.0 (7)
HFFr	2.125 (8)	1.0 (9)	3.167 (6)	2.8 (5)
Steatosis component of NAS^b^ (0–3)				
RC	0.125 (8)	0 (8)	0.1667 (6)	0.1429 (7)
HFFr	1.375 (8)	0.5556 (9)	1.833 (6)	1.8 (5)
Inflammatory component of NAS^c^ (0–3)				
RC	0.25 (8)	0.125 (8)	0.3333 (6)	0.8571 (7)
HFFr	0.75 (8)	0.4444 (9)	1.167 (6)	1 (5)
Ballooning component of NAS^d^ (0–2)				
RC	0 (8)	0 (8)	0 (6)	0 (7)
HFFr	0 (8)	0 (9)	0.1667 (6)	0 (5)
Fibrosis stage (0–3)^e^				
RC	0.14 (8)	0.07 (8)	0.20 (6)	0.51 (7)
HFFr	0.33 (8)	0.10 (9)	0.75 (6)	0.69 (5)

a NAS was estimated to be significantly higher in livers of HFFr-fed mice than in livers of RC-fed animals in both genotypes (Kruskal-Wallis test; *P* < .0001). DMSO-treated *Nrf2*^*+/+*^ mice fed the HFFr diet had a significantly higher NAS than did TBE-31-treated mice on the HFFr diet (Kruskal-Wallis H test; *P* < .05). NAS estimates in livers of *Nrf2*^*-/-*^ animals fed the HFFr diet were higher, but not significantly higher, than those in livers of *Nrf2*^*+/+*^ mice fed the HFFr diet (Kruskal-Wallis test; *P* > .05).

b Livers from mice fed the HFFr diet exhibited more steatosis than their RC-fed counterparts in both genotypes (Kruskal-Wallis H test; *P* < .0001). However, no significant difference in hepatic steatosis was observed between DMSO- and TBE-31-treated mice fed the same diet.

c The hepatic inflammatory component was significantly higher in mice fed the HFFr diet when compared with their RC-fed counterparts (Kruskal-Wallis H test; *P* < .0001). No significant difference in inflammation was observed between livers from DMSO- and TBE-31-treated mice fed on same diet in both genotypes.

d Ballooning was seen only in the liver of 1 *Nrf2*^*-/-*^ mouse that was fed the HFFr diet.

e Livers from mice fed the HFFr diet showed more fibrosis (this is not included in NAS calculation) when compared with mice fed the RC diet in both genotypes. *Nrf2*^*+/+*^ mice fed the HFFr diet exhibited significantly less hepatic fibrosis when treated with TBE-31 than did *Nrf2*^*+/+*^ mice treated with DMSO (Kruskal-Wallis H test; *P* < .05). Fibrosis in livers of *Nrf2*^*-/-*^ mice fed the HFFr diet was significantly higher than in livers of their wild-type counterparts (Kruskal-Wallis H test; *P* < .05). TBE-31 treatment had no effect on liver fibrosis in *Nrf2*^*-/-*^ mice.

### TBE-31 Requires Nrf2 to Suppress Steatosis, Endoplasmic Reticulum Stress, Inflammation, and Oxidative Stress in Livers From HF30Fr-Fed Mice

**Figure 16:**
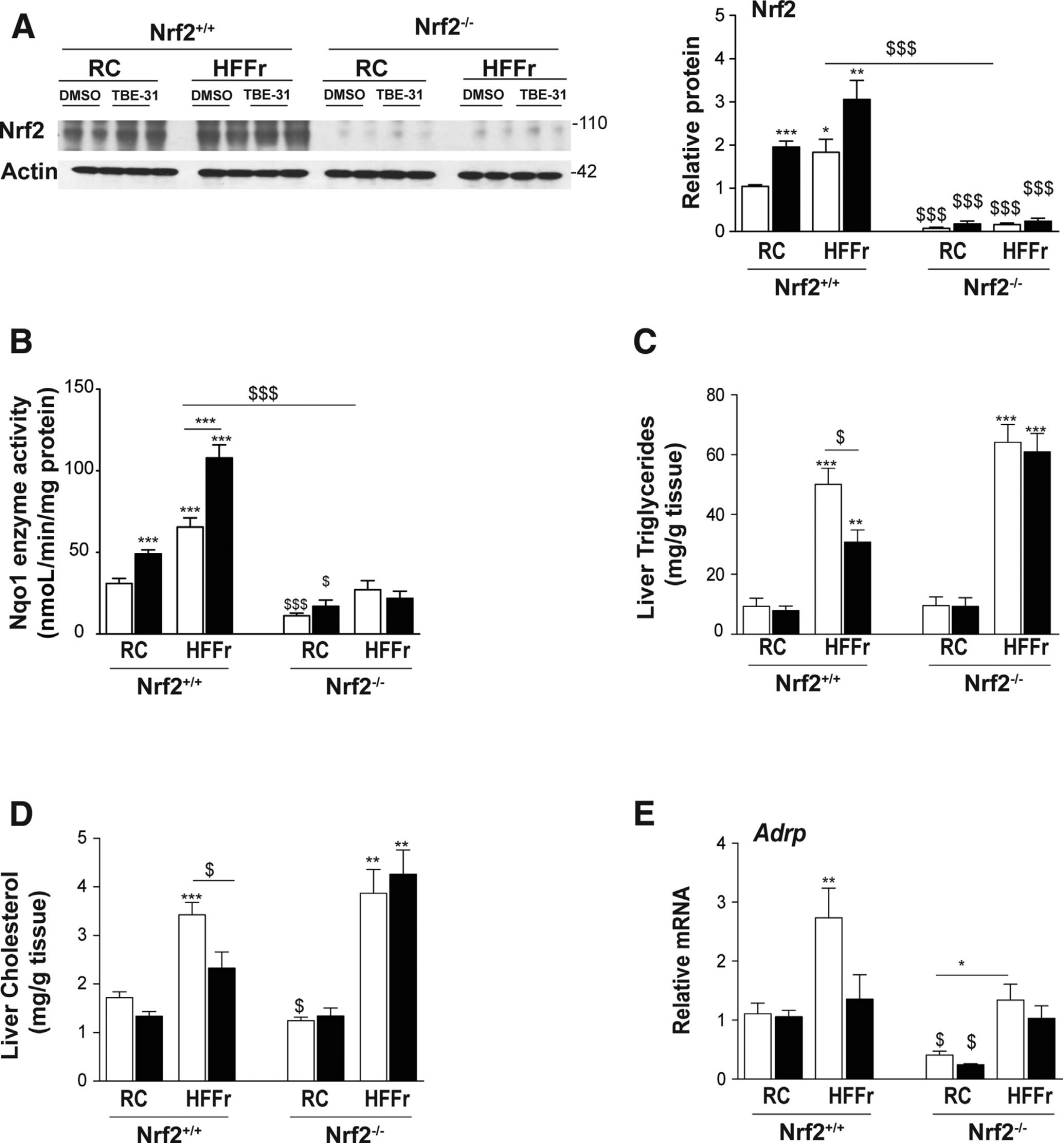
**TBE-31 does not decrease steatosis in the livers of HFFr-fed *Nrf2***^***-/-***^**mice.** After completion of Study 2, livers were removed from *Nrf2*^*+/+*^ and *Nrf2*^*-/-*^ mice to confirm absence of Nrf2 in the knockout mouse, and for biochemical analyses. (*A, left side*) Representative immunoblot of Nrf2 in livers of mice of both genotypes fed either a RC-diet or a HFFr-diet and treated with DMSO or TBE-31. (*A, right side*) Densitometric scans of the immunoblots (n = 4 biologic replicates). (*B*) Nqo1 catalytic activity in hepatic extracts from *Nrf2*^*+/+*^ and *Nrf2*^*-/-*^ mice fed RC or HFFr diets, and treated with DMSO or TBE-31. (*C, D*) Triglycerides and cholesterol in livers from *Nrf2*^*+/+*^ mice fed RC and HF30Fr diets are shown on the *left side* of the graphs, and results from *Nrf2*^*-/-*^ mice fed RC and HF30Fr diets are presented on the *right side*. (*E*) qRT-PCR for the lipid droplet-associated protein Adrp. In all cases, *white bars* represent DMSO and *black bars* represent TBE-31. In *A*, 4 mice per group were examined. In *B–D*, 6–8 mice per group. Data are means ± SEM. Significant differences from *Nrf2*^*+/+*^ control are indicated: ^∗,$^*P* < .05; ^∗∗^*P* < .01; ^∗∗∗,$$$^*P* < .001.

As expected, immunoblotting showed the 6-week TBE-31 treatment increased abundance of Nrf2 protein in livers of wild-type mice; by contrast, Nrf2 protein was not detected in livers of Nrf2-null mice under basal conditions or following TBE-31 treatment (Figure 16*A*). These Nrf2 immunoblotting data closely mirrored levels of Nqo1 enzyme activity in the same livers (Figure 16*B*). Collectively, these findings confirm that classic Nrf2-target genes are downregulated and cannot be induced by TBE-31 in livers of *Nrf2*^*-/-*^ mice.

**Figure 17:**
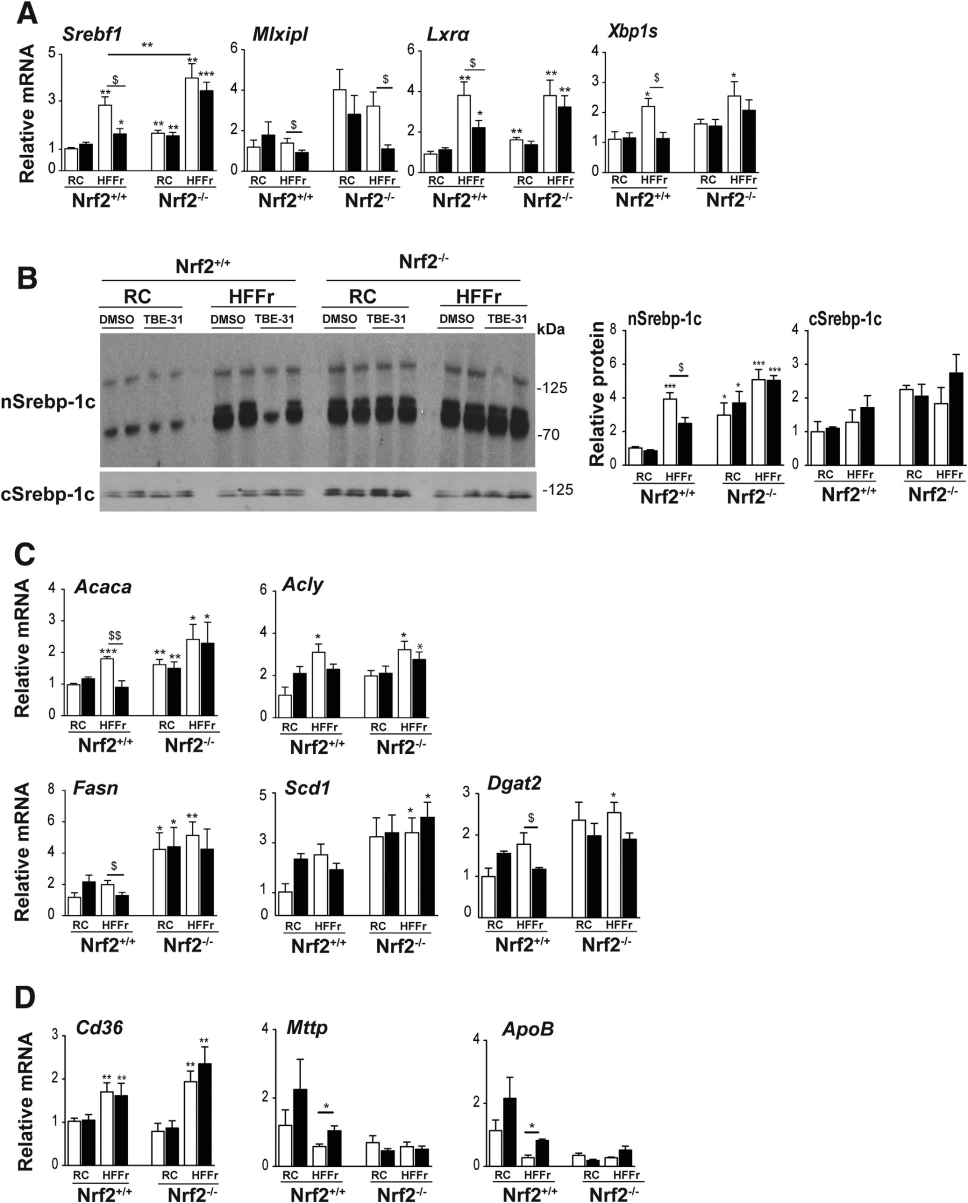
**TBE-31 does not decrease expression of lipogenic transcription factors or fatty acid synthesis enzymes in livers of HFFr-fed *Nrf2***^***-/-***^**mice.** The expression of transcription factors associated with lipid metabolism, and their target genes, were examined in livers from *Nrf2*^*+/+*^ and *Nrf2*^*-/-*^ mice that had been fed either a RC or HF30Fr (HFFr) diet and treated with DMSO or TBE-31. (*A*) qRT-PCR for the lipogenic transcription factors Srebf1, Mlxipl, Lxrα and Xbp1s. (*B*) Representative Srebp-1c immunoblots of cytoplasmic (cSrebp-1c) and nuclear (nSrebp-1c) protein on *left side*, with plots of densitometric scans on *right side* (n = 4 biologic replicates). (*C*) qRT-PCR for the fatty acid synthesis genes Acaca, Acly, Fasn, Scd1, and Dgat2. (*D*) qRT-PCR for the lipid import gene *Cd36* and the export genes *Mttp* and *ApoB*. *White bars*, DMSO treated; *black bars*, TBE-31 treated. In *A*, *C*, and *D*, 6–8 mice per group. In *B*, n = 4. Results are means ± SEM. Significant increases relative to that found in livers of RC-fed DMSO-treated *Nrf2*^*+/+*^ mice are indicated by: *P < .05; **P < .01; ***P < .001. Significant decreases relative to HF30Fr-fed DMSO-treated *Nrf2*^*+/+*^ mice are indicated: ^$^*P* < .05; ^$$^*P* < .01.

Although TBE-31 treatment of HF30Fr-fed *Nrf2*^*+/+*^ mice decreased total liver triglycerides and cholesterol, this was not observed in HF30Fr-fed Nrf2-null mice (Figure 16*C*, *D*). Similarly, TBE-31 did not decrease expression of the lipid droplet marker protein Adrp in livers of HF30Fr-fed Nrf2-null mice (Figure 16*E*). Although TBE-31 suppressed the increase in expression of *Srebf1*, *Lxrα*, and *Xbp1s* in livers of *Nrf2*^*+/+*^ mice fed the HF30Fr diet, it was unable to do so in livers of HF30Fr-fed Nrf2-null mice (Figure 17*A*), suggesting that the ability of TBE-31 to suppress expression of these transcription factors is mediated by Nrf2. Consistent with the qRT-PCR data for *Srebf1*, immunoblotting revealed TBE-31 blunted the increase in nuclear Srebp-1c protein in livers of HF30Fr-fed *Nrf2*^*+/+*^ mice, but did not do so in livers of HF30Fr-fed *Nrf2*^*-/-*^ mice (Figure 17*B*). We also noted that the decrease in mRNA for Lxrα in livers of HF30Fr-fed mice treated with TBE-31 was not associated with an obvious reciprocal increase in mRNA for Fxr and Shp in either *Nrf2*^*+/+*^ or *Nrf2*^*-/-*^ genotypes (data not shown), suggesting that the Nrf2-dependent suppression of Lxrα does not involve Fxr or Shp. Surprisingly, TBE-31 decreased mRNA for Chrebp, encoded by *Mlxipl*, in livers of both HF30Fr-fed *Nrf2*^*+/+*^ and *Nrf2*^*-/-*^ mice (Figure 17*A*), a result that indicates it can antagonize Chrebp through a mechanism that does not involve Nrf2. Consistent with the qRT-PCR results for *Srebf1*, *Lxrα*, and *Xbp1s*, TBE-31 did not downregulate mRNA for the fatty acid and triglyceride synthesis enzymes Acaca, Fasn, and Dgat2 in livers of HF30Fr-fed *Nrf2*^*-/-*^ mice (Figure 17*C*). Although the HF30Fr diet increased mRNA for the lipid importer Cd36 in livers of *Nrf2*^*+/+*^ and *Nrf2*^*-/-*^ mice, TBE-31 had no additional effect on *Cd36* expression in livers of either *Nrf2*^*+/+*^ or *Nrf2*^*-/-*^ mice (Figure 17*D*). Lastly, although TBE-31 increased mRNA levels for Mttp and ApoB, which are involved lipoprotein assembly, in livers of *Nrf2*^*+/+*^ mice, it did not increase mRNA for these proteins in livers of *Nrf2*^*-/-*^ mice (Figure 17*D*).

**Figure 18:**
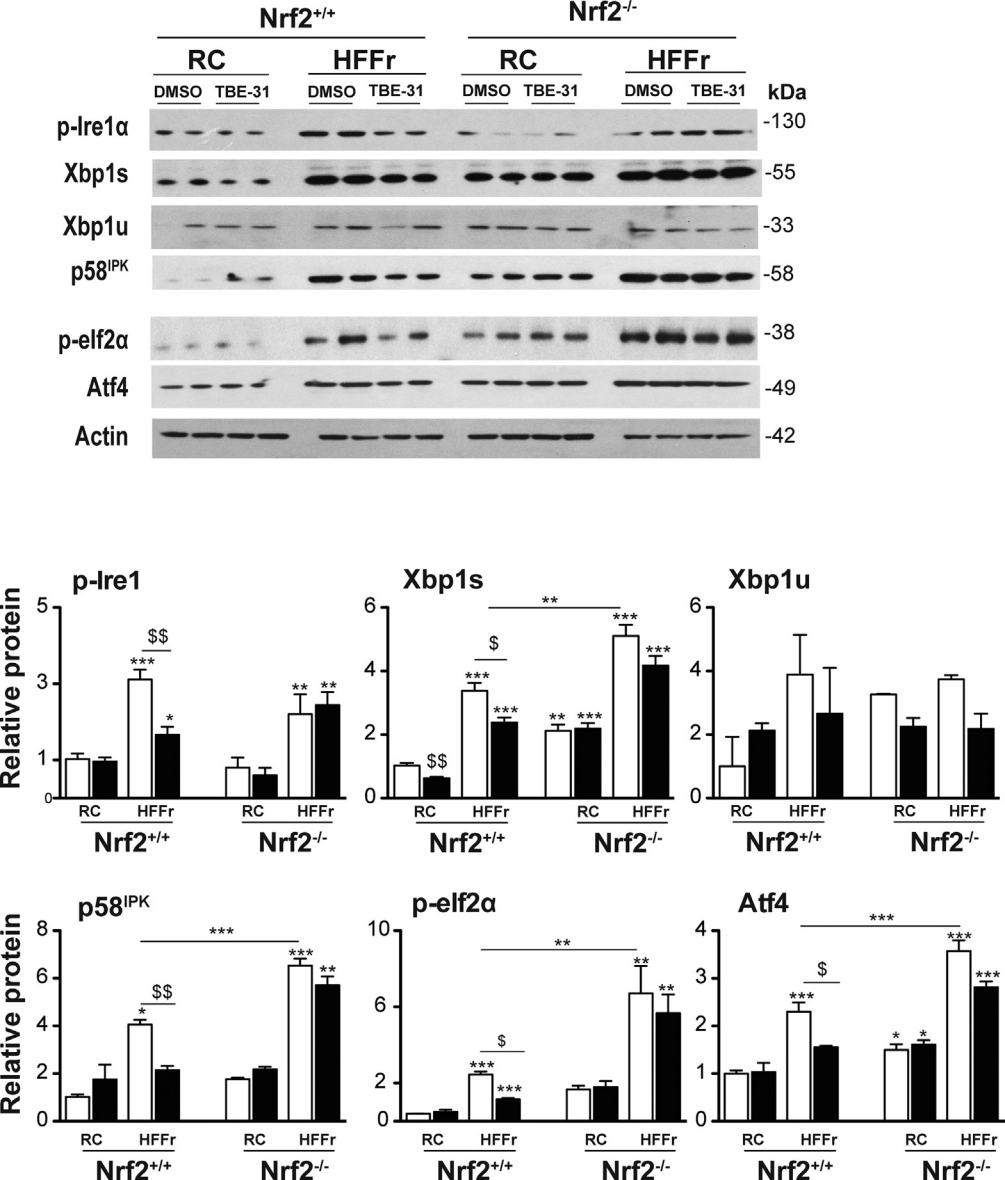
**TBE-31 fails to suppress ER stress in livers of HFFr-fed *Nrf2***^***-/-***^**mice.** Livers were collected from *Nrf2*^*+/+*^ and *Nrf2*^*-/-*^ mice at the end of Study 2, and proteins and genes involved in the UPR were examined. Representative immunoblots of p-Ire1α, Xbp1s and Xbp1u, p58^IPK^, p-eIf2α and Atf4 proteins, along with actin as a loading control, are shown at the *top*. Plots of densitometric scans from the blots are shown at the *bottom* (n = 4 biologic replicates). *White bars*, DMSO treated; *black bars*, TBE-31 treated. Results are means ± SEM. Significant increases relative to that found in livers of RC-fed *Nrf2*^*+/+*^ mice are indicated by: *P < .05; **P < .01; ***P < .001. Significant decreases relative to HF30Fr-fed *Nrf2*^*+/+*^ mice are indicated: ^$^*P* < .05; ^$$^*P* < .01.

Immunoblotting suggested that at least 2 arms of the UPR were activated in livers of RC-fed *Nrf2*^*-/-*^ mice when compared with livers of RC-fed *Nrf2*^*+/+*^ mice, as evidenced by increases in Xbp1s (downstream of Ire1α), and Atf4 (downstream of Perk) (Figure 18). This also revealed livers from HF30Fr-fed *Nrf2*^*+/+*^ mice contained increased levels of the ER marker proteins p-Ire1α, Xbp1s, p58^IPK^, p-eIf2α, and Atf4 that was attenuated by treatment with TBE-31. By contrast, TBE-31 did not decrease these ER marker proteins in livers from HF30Fr-fed *Nrf2*^*-/-*^ mice.

**Figure 19:**
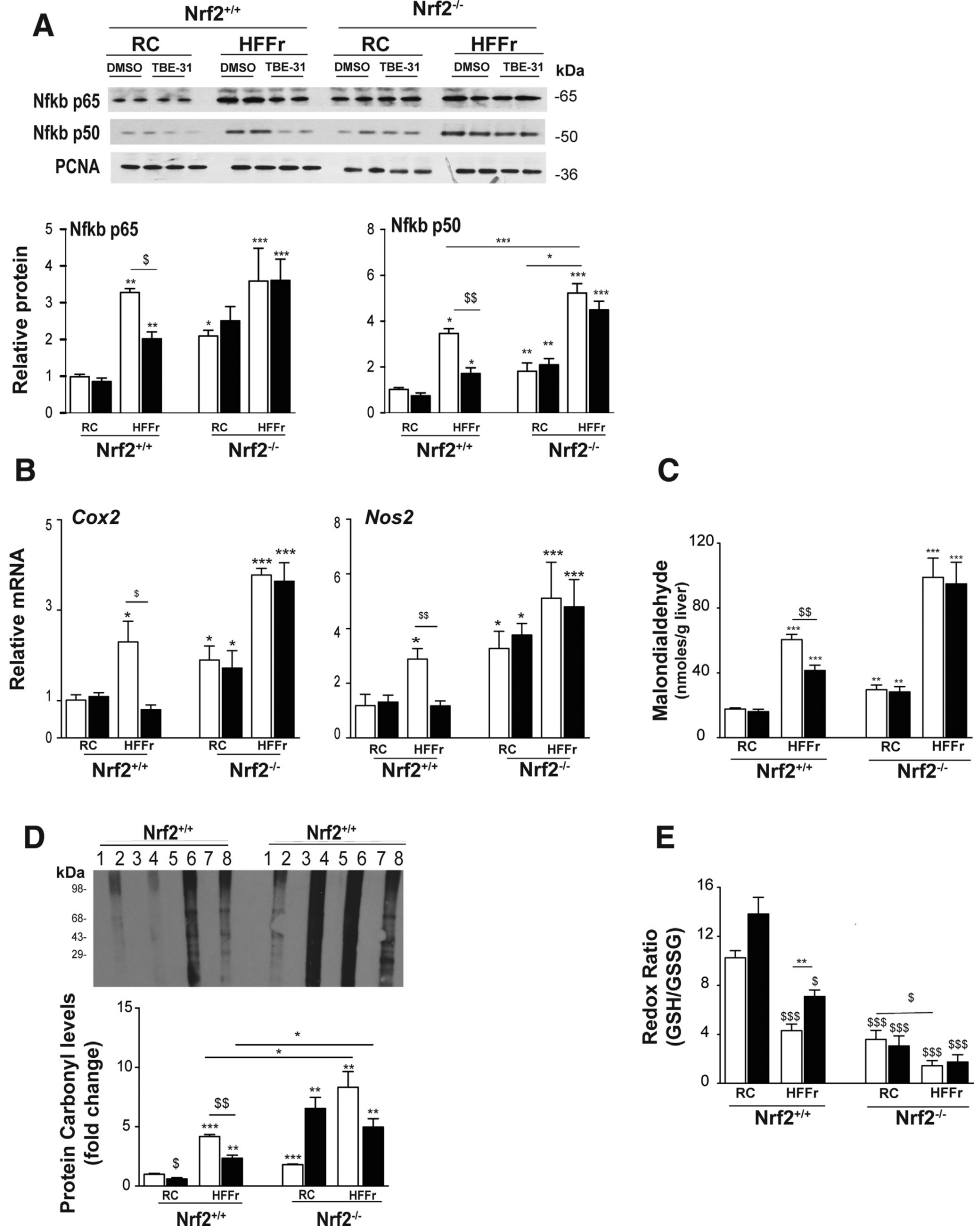
**TBE-31 fails to suppress inflammation and oxidative stress in HFFr-fed *Nrf2***^***-/-***^**mouse liver.** The extent of inflammation and oxidative stress in livers of *Nrf2*^*+/+*^ and *Nrf2*^*-/-*^ mice at the end of Study 2 was examined. (*A*) Representative immunoblots of hepatic nuclear levels of Nfkb p65 and Nfkb p50 (using proliferating cell nuclear antigen as a loading control), along with densitometric scans (n = 4 biologic replicates), from *Nrf2*^*+/+*^ and *Nrf2*^*-/-*^ mice fed RC or HFFr diets and treated with DMSO or TBE-31. (*B*) qRT-PCR for the Nfkb-target genes *Cox2* and *Nos2* from the same livers (6–8 mice per group). (*C*) Malondialedehyde levels in livers from *Nrf2*^*+/+*^ and *Nrf2*^*-/-*^ mice (6–8 mice per group). (*D*) Oxidized protein levels in livers of *Nrf2*^*+/+*^ and *Nrf2*^*-/-*^ mice shown as a representative Oxyblot, with densitometric quantification below: lanes 1, 3, 5, and 7 negative controls; 2, RC-fed DMSO; 4, RC-fed TBE-31; 6, HF30Fr-fed DMSO; 8, HF30Fr-fed TBE-31 (n = 4). (*E*) Ratio of GSH to GSSG (6–8 mice per group). In all cases, *white bars* represent DMSO and *black bars* represent TBE-31. Data are means ± SEM. Significant differences are signified: ^∗,$^*P* < .05; ^∗∗,$$^*P* < .01; ^∗∗∗,$$$^*P* < .001.

Immunoblotting revealed that the HF30Fr diet stimulated nuclear accumulation of Nfkb p65 and p50 in livers of *Nrf2*^*+/+*^ and *Nrf2*^*-/-*^ mice, and that although TBE-31 could attenuate this in livers of wild-type mice it was unable to do so in *Nrf2*^*-/-*^ livers (Figure 19*A*). qRT-PCR of mRNAs for Cox2 and Nos2 indicated that the HF30Fr diet stimulated expression of proinflammatory genes in the livers of *Nrf2*^*+/+*^ and *Nrf2*^*-/-*^ mice but, whereas TBE-31 blunted this increase in wild-type mice, TBE-31 was unable to do so in the mutant mice (Figure 19*B*).

As anticipated, the inability of TBE-31 to suppress NASH in *Nrf2*^*-/-*^ mice was associated with higher levels of hepatic malondialdehyde, oxidized protein, and GSSG than in *Nrf2*^*+/+*^ mice (Figure 19*C*–*E*) suggesting that TBE-31 did not suppress oxidative stress caused by the HF30Fr diet in *Nrf2*^*-/-*^ mice.

## Discussion

We have examined whether pharmacologic activation of Nrf2 using the acetylenic tricyclic bis(cyano enone) compound TBE-31 can suppress NASH in mice in which diet-stimulated metabolic disease had already been established, reflecting the situation encountered in clinical practice. The experiments described herein reveal that treatment of mice with TBE-31 conferred multiple metabolic benefits insofar as it increased whole-body insulin sensitivity and improved glucose homeostasis (better glucose disposal and reduced gluconeogenesis). Moreover, within the liver, TBE-31 markedly diminished steatosis and inflammation (ie, NASH, as evidenced by the NAS calculations) and fibrosis (which is not included in NAS calculations). Most importantly, our data show that TBE-31 requires the presence of Nrf2 to produce these beneficial pleiotropic effects.

### Influence of Nrf2 Activation on Glucose Homeostasis

It is known that *Nrf2*^*-/-*^ mice are more sensitive than *Nrf2*^*+/+*^ mice to streptozotocin-induced diabetes, and that pharmacologic activation of Nrf2 with sulforaphane (SFN) can inhibit the development of diabetes,^46^, ^47^ but it is not known whether activation of Nrf2 in wild-type mice after diabetes has been induced by physiological stimuli (ie, chronic overnutrition) can mitigate disease. Uniquely, in the present study, we have demonstrated that pharmacologic activation of Nrf2 can reverse type 2 diabetes by performing metabolic analyses before and after 4−5 weeks therapeutic intervention with TBE-31 in adult *Nrf2*^*+/+*^ mice fed a HFFr diet, thereby providing clear evidence that the acetylenic tricyclic bis(cyano enone) can improve glucose disposal and insulin sensitivity. Our finding that TBE-31 treatment reverses insulin resistance in mice that have been fed chronically an HFFr diet is consistent with a report that treatment of 6-week-old diabetic *db/db* mice with the Nrf2 activator CDDO-Im for 10 weeks increased glucose clearance and lowered plasma insulin levels during an oral GTT.^31^ However, our results are at variance with a recent report that 4-week treatment of adult C57BL/6J mice that had received a 60% HF diet for 10 weeks with the Nrf2 activator SFN improved glucose tolerance during an intraperitoneal GTT but did not improve insulin sensitivity.^48^ In the present investigation, changes in glucose homeostasis affected by TBE-31 were monitored in Study 1 using ITT and GTT, and in Study 2 using ITT and PTT. None of these provide a reference method for defining insulin resistance, because this is only achieved using hyperinsulinemic euglycemic clamps. Therefore, further work is required to determine whether the difference in the ability of TBE-31 and SFN to improve insulin sensitivity is caused by methodological factors (eg, the type of diet, duration of feeding, potency, or pharmacokinetic properties of Nrf2 activators).

The fact that *Nrf2*^*-/-*^ mice fed a HFFr diet became insulin resistant (as assessed by ITT) is surprising because we have previously reported that the knockout mice fed an HF diet exhibit better insulin sensitivity than HF-fed *Nrf2*^*+/+*^ mice.^24^ This outcome, along with worsened NASH, displayed by HFFr-fed *Nrf2*^*-/-*^ mice, suggests Nrf2 plays a previously unrecognized role in protection against fructose-driven metabolic disorder. The reasons for this are not known. Possibly overconsumption of fructose may cause insulin resistance by increasing hepatic diacylglycerol levels and activating protein kinase Cε.^49^ Clearly, this issue warrants further investigation.

### Activation of Nrf2 Attenuates Hepatic Lipid Metabolism

In the present study, Nrf2-mediated inhibition of HFFr-stimulated liver steatosis by TBE-31 involves positive and negative mechanisms. On the one hand, TBE-31 increased, in a Nrf2-dependent manner, the abundance of mRNA for proteins that limit lipid accumulation in the liver, such as those involved in fatty acid oxidation (eg, Acox2, Ces1g, Cpt1a, and Scad) and export of triglycerides (eg, Mttp and ApoB). On the other hand, TBE-31 decreased, in a Nrf2-dependent manner, mRNA for Srebp-1c, Xbp1s, and Lxrα, transcription factors that orchestrate *de novo* lipogenesis and have been implicated in hepatic steatosis.^49^, ^50^

Among the previously mentioned lipogenic transcription factors, Srebp-1c and Xbp1s are integrated into the UPR and are activated by ER stress.^42^ Because evidence suggests Nrf2 marshals the expression of genes that collectively antagonize ER stress,^24^ we propose that activation of Nrf2 by TBE-31 blunts ER stress caused by the HFFr diet, and this in turn decreases the activity of Srebp-1c and Xbp1s, along with expression of their target genes (eg, *Acaca, Acly*, *Dgat2*, *Fasn*, and *Scd1*). Our hypothesis that TBE-31 protects against excessive hepatic steatosis, at least in part, by blunting ER stress, could be likened to protection against the toxic effect of the ER stressor tunicamycin by the Nrf2 activator *3H-*1,2-dithiole-3-thione,^51^ or protection conferred by knockdown of Keap1 against the ER stress-mediated apoptotic effects of alkylating agents.^52^

Besides downregulating Srebp-1c and Xbp1s, we found pharmacologic activation of Nrf2 is associated with a decrease in abundance of mRNA for Lxrα. However, by contrast with Srebp-1c and Xbp1s, Lxrα is not activated by ER stress,^43^ and so some other mechanism must be involved. It is possible that the ability of Nrf2 to downregulate the expression of *Lxrα* is a consequence of Nrf2 decreasing the levels of endogenous oxysteroids: this reasoning is based on the fact that Lxrα is a nuclear receptor that is activated by oxysteroid ligands, and that the human *LXRα* gene autoregulates itself because it contains an LXR response element (LXRE) in its promoter region^53^, ^54^; it is, however, unclear if this mechanism is relevant to the mouse because there is controversy about whether the putative LXRE in the promoter region of mouse *Lxrα* is functional.^55^, ^56^ An additional possibility is that activation of Nrf2 downregulates expression of *Lxrα* because it decreases the hepatic levels of thyroid hormone: this suggestion is based on the report that triiodothyronine induces expression of *Lxrα* in mouse liver via the thyroid hormone receptor.^56^ Another explanation is that activation of Nrf2 antagonizes Lxrα activity, and hence its expression, by facilitating inhibitory phosphorylation of Lxrα at one of several Thr residues by adenosine monophosphate-activated protein kinase.^57^ An alternative possibility proposed by Kay et al^44^ is that activation of Nrf2 downregulates *Lxrα* expression because it leads to induction and deacetylation of Fxr that results in induction of Shp protein, which in turn blunts expression of the *Lxrα* gene by forming a nonproductive heterodimer with Lxrα. However, this hypothesis was based on experiments using SFN to activate Nrf2, and we think this is unlikely in the case of TBE-31 because although expression of *Shp* was lower in livers of *Nrf2*^*-/-*^ mice than those of *Nrf2*^*+/+*^ mice, the expression of *Fxr* did not differ in livers from either genotype, nor was the mRNA for Shp or Fxr inducible by TBE-31 in livers of *Nrf2*^*+/+*^ mice. Clearly, further work is required to determine the mechanisms by which Nrf2 represses Lxrα.

It remains unclear how TBE-31 antagonizes the expression of Chrebp. Most importantly, we found TBE-31 attenuated the expression of Chrebp in livers of *Nrf2*^*-/-*^ mice, indicating that the acetylenic tricyclic bis(cyano enone) exerts Nrf2-independent effects. This action of TBE-31 possibly reflects changes in insulin sensitivity because the abundance of Chrebp mRNA responds dramatically to changes in insulin levels in human differentiated preadipocytes.^58^ Further work is required to address this issue.

### Influence of Nrf2 Activation on Hepatic Inflammation and Nonalcoholic Steatohepatitis

We found Nrf2-mediated suppression of liver inflammation during the development of NASH involves blunting of activation of AP-1 and Nfkb. It is unclear in our murine model whether ER stress or oxidative stress is more influential in stimulating activation of AP-1 and Nfkb by the HFFr diet, but because both forms of stress are interrelated^59^, ^60^ it is plausible that pharmacologic activation of Nrf2 by TBE-31 diminishes activation of AP-1 and Nfkb by countering ER and oxidative stress. TBE-31 decreased Il-1β mRNA levels, in line with the proposal that Nrf2 inhibits transcription of the *Il-1β* gene by binding to the promoter and preventing recruitment of RNA pol II.^61^ Therefore, it is likely TBE-31 increases the effectiveness of this mode of repression of inflammation. Furthermore, TBE-31 likely inhibits recruitment of monocytes and neutrophils into the liver because it suppressed mRNA levels for Mcp1, and for elastase and myeloperoxidase.

The demonstration that activation of Nrf2 by TBE-31 ameliorates NASH is consistent with several earlier reports in which pharmacologic activators of Nrf2 including baicalein,^62^ SFN,^63^ or Ezetimibe,^64^ or genetic activation of Nrf2 by expression of a hypomorphic *Keap1* allele^65^ or hepatocyte-specific knockout of Keap1,^66^ inhibit NASH in rodents caused by an MCD diet or an HF diet. In all these previous experiments, however, the inducing agents were coadministered with the MCD/HF diet, or the genetic manipulation was constitutive, and therefore they do not address the question of whether Nrf2 can ameliorate established disease. Thus, our present findings add substantially to those in the literature by showing TBE-31−treatment can reverse insulin resistance and, by implication, also NASH.

### Influence of Nrf2 Activation on Liver Fibrosis

Our discovery that TBE-31 can inhibit liver fibrosis in HFFr-fed mice with NASH in an Nrf2-dependent manner is particularly noteworthy. Specifically, we found that histologic evidence of fibrosis and expression of *Col1a1* and *α-Sma* were substantially diminished in HFFr-fed wild-type mice treated with TBE-31, when compared with DMSO-treated mice. These results are in good agreement with a previous study in rats in which the Nrf2 activators oltipraz and NK-252 attenuated progression of NASH-related fibrosis stimulated by a choline-deficient L-amino acid-defined diet,^67^ but whether or not the antifibrosis effects of oltipraz and NK-252 were mediated by Nrf2 was not addressed.

### Influence of Endoplasmic Reticulum Stress and Cirrhosis on Nrf2

Wu et al^68^ have demonstrated that during end-stage liver cirrhosis in mice, hepatic Nrf2 is suppressed as a consequence of activation of the Ire1α-Xbp1 arm of the UPR, thereby exacerbating oxidative stress and promoting further disease. Specifically, these workers reported that experimental liver cirrhosis, caused by CCl_4_, resulted in increases in Xbp1s and its downstream target Hrd1, which in turn stimulated ubiquitylation and degradation of Nrf2, thereby decreasing expression of *Nqo1* and *Gclm*. In the present study, we discovered chronic feeding of *Nrf2*^*+/+*^ mice with the HFFr diet stimulated ER stress in the liver, with clear evidence of activation of the Xbp1 arm (Figure 10*A* and *B*). However, HFFr-feeding had a mixed effect on expression of hepatic Nrf2-target genes insofar as the diet increased expression of *Gclc*, *Gclm*, *Hmox1*, and *Slc7a11*, while decreasing the expression of *Txnrd1*, *Cat*, and *Prdx6* (Figure 13*D*). Importantly, we found that treatment of HFFr-fed mice with TBE-31 increased the expression of all these Nrf2-target genes. Collectively, our findings suggest that stimulation of ER stress by chronic consumption of an HFFr diet is not sufficient to cause Hrd1-mediated suppression of Nrf2 that cannot be overcome by administration of TBE-31. Further work is required to define at what stage during the progression of NASH through to cirrhosis is Nrf2 suppressed by Hrd1, and what additional environmental and/or biochemical factors, besides just ER stress, are required.

## Conclusions

**Figure 20:**
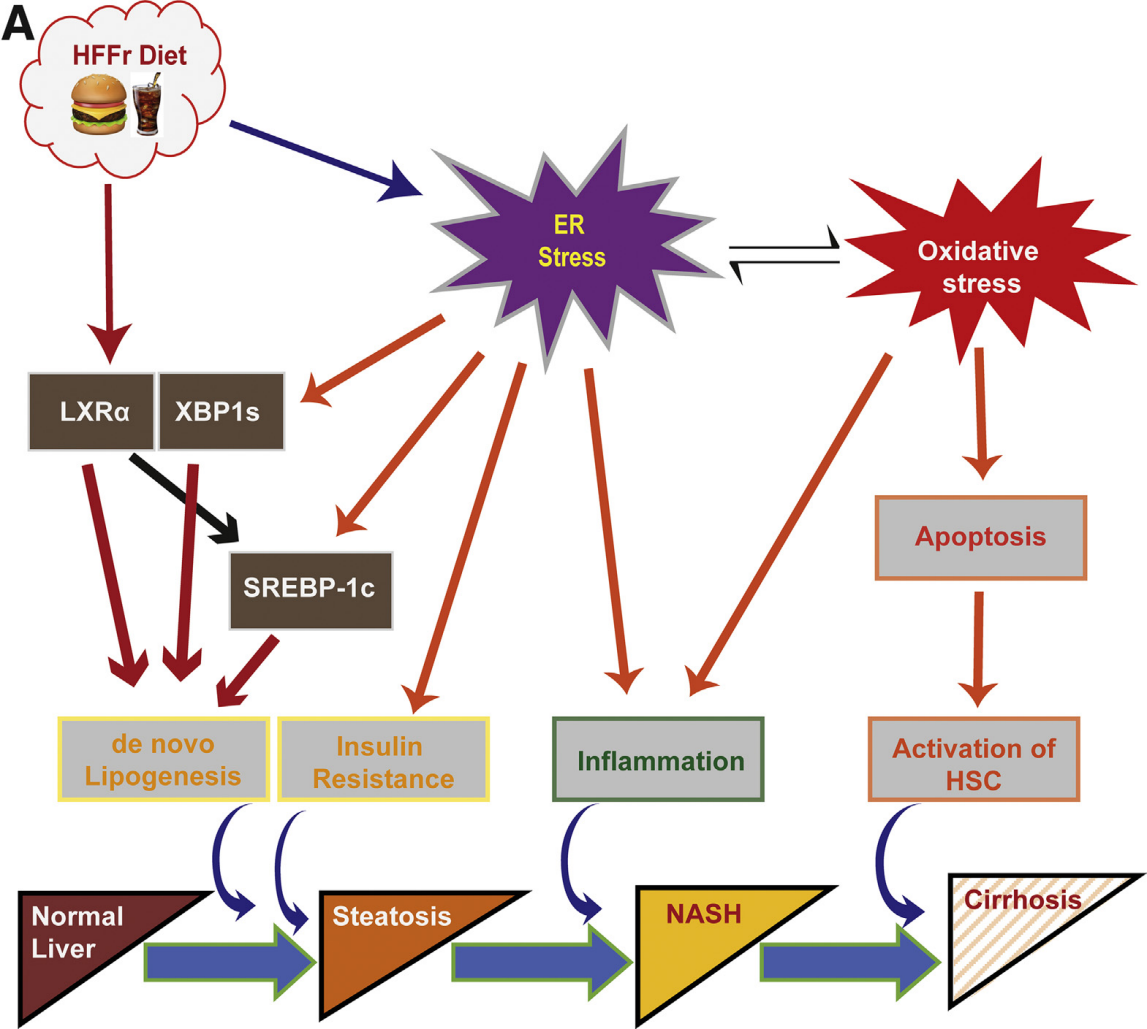

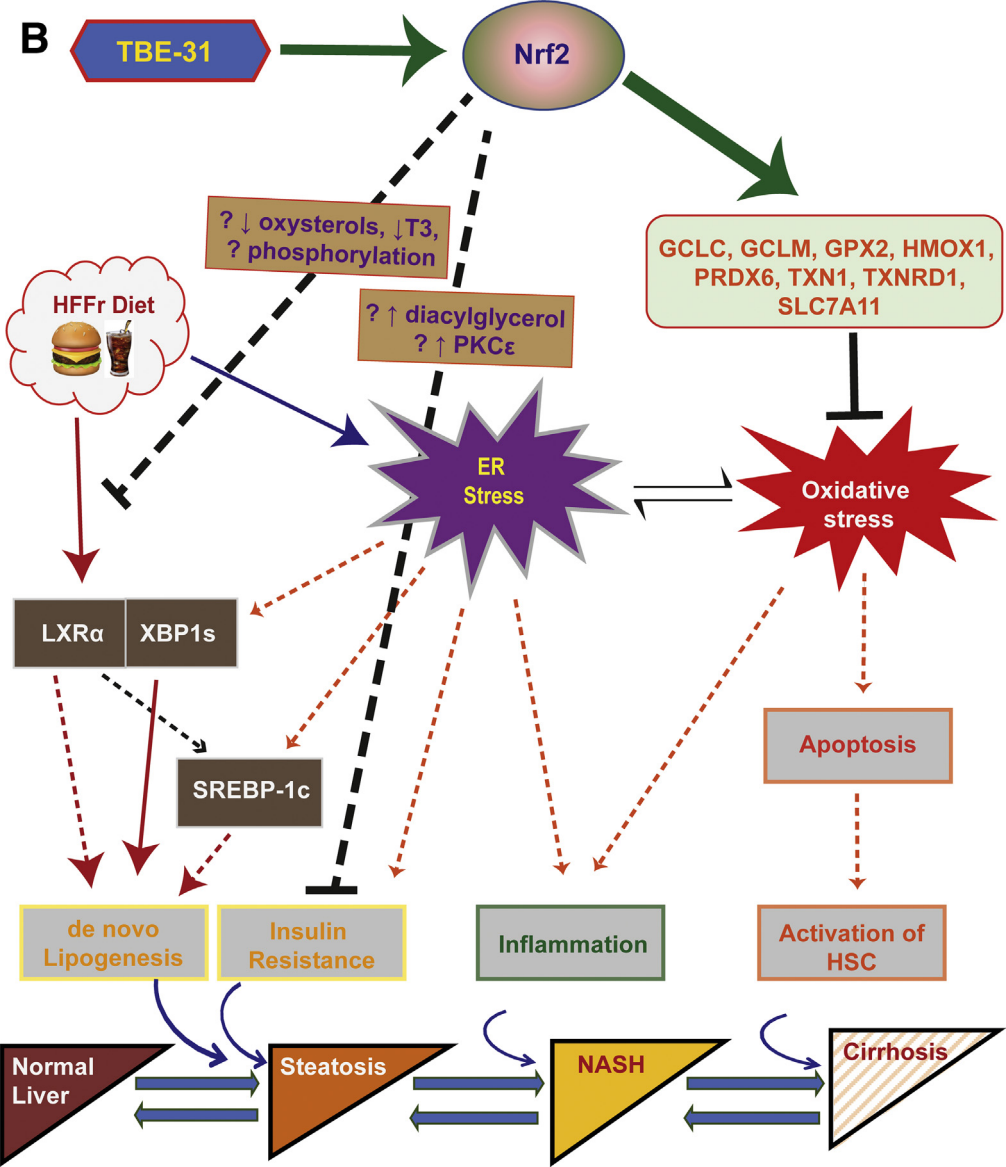
**Nrf2-dependent mechanisms by which TBE-31 suppresses insulin resistance, NASH and cirrhosis.** (*A*) The various mechanisms by which consumption of a high-fat and high-fructose diet is believed to drive hepatic steatosis, NASH, and cirrhosis.^4^, ^5^, ^8^ (*B*) The processes by which we envisage pharmacologic activation of Nrf2 inhibits and/or reverses liver disease caused by consumption of a diet enriched with high-fat and high-fructose foodstuffs. For further information see Results and Discussion sections. HSC, hepatic stellate cell.

This investigation shows TBE-31 provides an effective therapeutic strategy to treat NASH through the ability of Nrf2 to direct changes in gene expression that antagonize lipogenesis, ER stress, inflammation, oxidative stress, and fibrosis (summarized in Figure 20). Work is now required to establish whether pharmacologic activation of Nrf2 in the human can improve metabolic syndrome, NASH, and liver fibrosis. Such clinical studies are entirely feasible because Nrf2 activators have already been demonstrated to improve diabetes-related biomarkers.^48^
